# Electrochemical nitrite sensing for urine nitrification

**DOI:** 10.1016/j.wroa.2020.100055

**Published:** 2020-05-23

**Authors:** Livia Britschgi, Kris Villez, Peter Schrems, Kai M. Udert

**Affiliations:** a Eawag, Swiss Federal Institute of Aquatic Science and Technology, 8600, Dübendorf, Switzerland; b IPS Elektroniklabor GmbH & Co. KG, 64839, Münster, Germany; c ETH Zürich, Institute of Environmental Engineering, 8093, Zürich, Switzerland

**Keywords:** Continuous measurement, In-situ measurement, Electrochemical measurement, Amperometric sensor, Critical nitrite concentration, Nitrite measurement

## Abstract

Sensing nitrite in-situ in wastewater treatment processes could greatly simplify process control, especially during treatment of high-strength nitrogen wastewaters such as digester supernatant or, as in our case, urine. The two technologies available today, i.e. an on-line nitrite analyzer and a spectrophotometric sensor, have strong limitations such as sample preparation, cost of ownership and strong interferences. A promising alternative is the amperometric measurement of nitrite, which we assessed in this study. We investigated the sensor in a urine nitrification reactor and in ex-situ experiments. Based on theoretical calculations as well as a practical approach, we determined that the critical nitrite concentrations for nitrite oxidizing bacteria lie between 12 and 30 mg_N_/L at pH 6 to 6.8. Consequently, we decided that the sensor should be able to reliably measure concentrations up to 50 mg_N_/L, which is about double the value of the critical nitrite concentration. We found that the influences of various ambient conditions, such as temperature, pH, electric conductivity and aeration rate, in the ranges expected in urine nitrification systems, are negligible. For low nitrite concentrations, as expected in municipal wastewater treatment, the tested amperometric nitrite sensor was not sufficiently sensitive. Nevertheless, the sensor delivered reliable measurements for nitrite concentrations of 5–50 mg_N_/L or higher. This means that the amperometric nitrite sensor allows detection of critical nitrite concentrations without difficulty in high-strength nitrogen conversion processes, such as the nitrification of human urine.

## Highlights

• An amperometric sensor allows reliable on-line measurement of nitrite.
• The sensor is suitable for process control in urine nitrification.
• The calibration curves are linear in a range of 0 to 50 mg_N_/L.
• Monthly calibration is sufficient to correct for sensor drifts.
• The sensor can be calibrated in-situ by triggering a short-term nitrite increase.

## Graphical abstract

**Figure:**
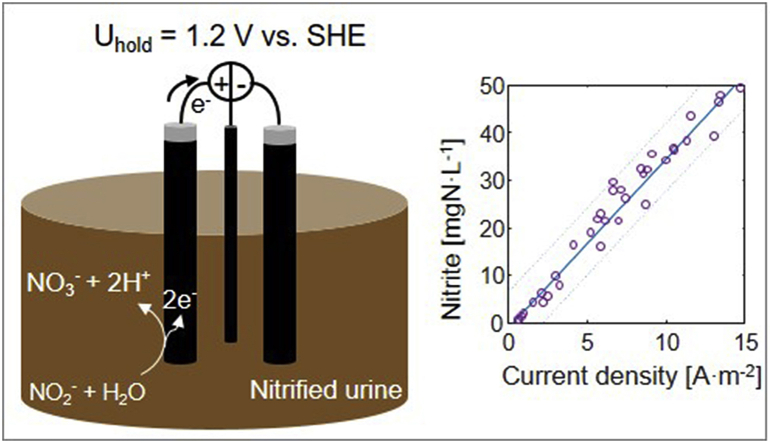

## 1 Introduction

In many wastewater treatment processes, maintaining low nitrite concentrations is crucial for several reasons. First, the effluent discharge limit for the nitrite concentration must be met. In Switzerland it lies at 0.3 mg_N_/L (Gujer, 2006) due to its toxicity for fish (Kroupova et al., 2005). Second, high nitrite concentrations must be avoided in order to prevent the formation of harmful gases such as nitric oxide and nitrous oxide during nitrification and denitrification (Schreiber et al., 2012). Third, nitrite is an intermediate product in nitrification and exists in equilibrium with its protonated form, free nitrous acid (HNO_2_), which can inhibit bacteria in wastewater treatment (Zhou et al., 2011). In many processes, such as conventional nitrification, for example nitrification of urine for fertilizer recovery, such inhibition should be avoided. Indeed, once nitrite oxidizing bacteria (NOB) are inhibited by nitrite, the compound accumulates faster and NOB are inhibited even more strongly, which can be detrimental for processes relying on complete nitrification of ammonia over nitrite to nitrate (Udert and Wächter, 2012). In a number of specialized processes, such as SHARON-Anammox (Volcke et al., 2006), DAEMON (Wett et al., 2007), and OLAND (Kuai and Verstraete, 1998), it is paramount that NOB growth is limited. The presence of nitrite can cause such growth, in turn leading to reduced energy and electron donor efficiency of these processes. Thus, nitrite is a key variable for optimal operation of many nitrifying processes, regardless of the desirability of NOB activity. An on-line nitrite measurement would be an ideal tool to detect detrimental nitrite concentration levels, and to prompt timely for corrective actions.

To our knowledge, two principles are available today to measure nitrite on-line in wastewater treatment. One is based on a colorimetric measurement, i.e. on-line nitrite analyzers (e.g. Nitrite analyzer Liquiline System CA80NO, Endress + Hauser, Reinach, Switzerland; SA9101, Skalar Analytical B.V., Breda, Netherlands). This technique shows no significant drift and has a high sensitivity and specificity. However, the cost of ownership (Ellram, 1995) of an on-line analyzer is typically high due to high hardware costs and intensive maintenance requirements (Mašić et al., 2015; Rieger et al., 2008). Furthermore, it is an ex-situ measurement and needs sampling preparation (Rieger et al., 2004). The complexity and costs for such automated sampling preparation can be an issue especially for small treatment plants.

Another continuous nitrite measurement is based on the light absorbance measurement principle (Thürlimann et al., 2017)*.* This is a spectrophotometric measurement, i.e. through measurement of light absorbance at multiple wavelengths, and makes use of the fact that many chemical compounds, including nitrite, absorb light differently at different wavelengths. Robust devices built around this principle are available commercially. Importantly, these measurements can be obtained in-situ and do not require sensitive sample preparation steps (Drolc and Vrtovšek, 2010). This type of sensors is sensitive to a variety of inorganic and organic compounds so that one can use a single device to measure multiple variables at once. Unfortunately, this also means that the absorbance measurements lack specificity. This, in turn, challenges accurate calibration and induces signal drift when the mixture of non-target components that absorb light, e.g., organic matter or solids, deviates from the mixtures observed during sensor calibration. Such a phenomenon is often described as a change of the background spectrum (Thürlimann et al., 2019). While sensor drift can be compensated by fault-tolerant control schemes (Deshpande et al., 2009; Thürlimann et al., 2019), it is generally considered desirable to avoid sensor drift entirely.

**Fig. 1:**
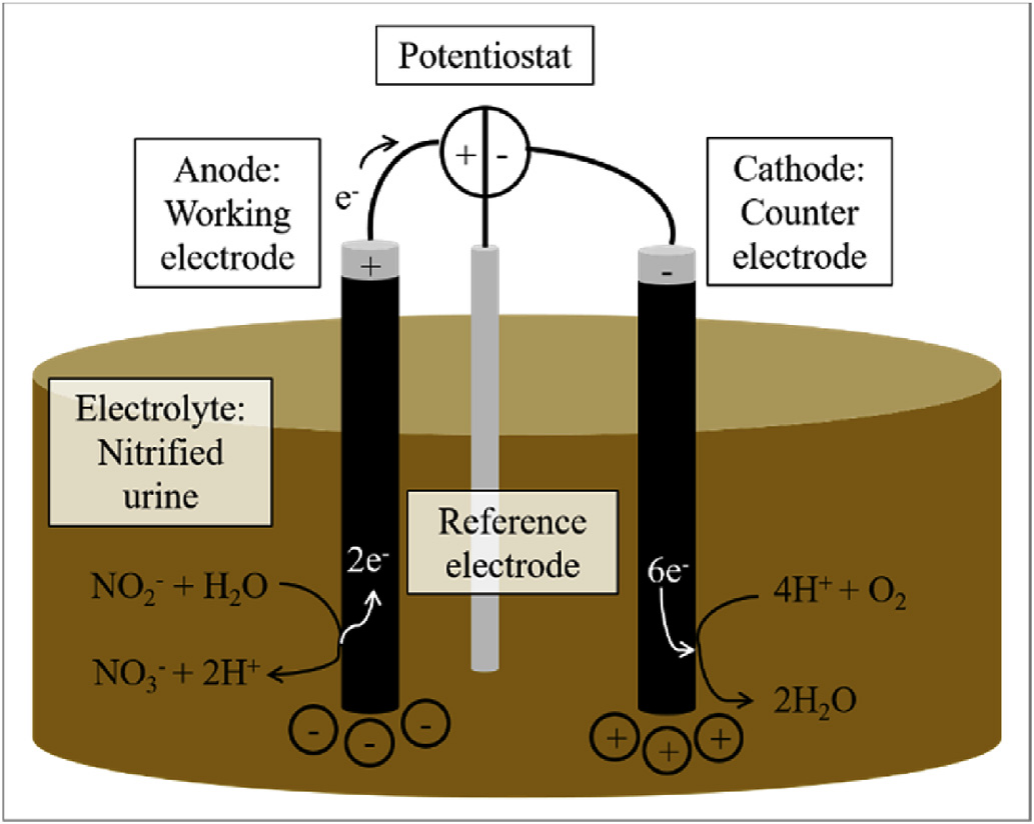
Scheme of the electrochemical nitrite measurement.

The amperometric nitrite measurement, which we assessed in this study, is a promising alternative that may lead to the construction of a drift-free on-line nitrite sensor. With this method, nitrite concentrations in aqueous solutions are measured electrochemically. The sensor consists of three electrodes: a reference electrode, a working electrode, in our case the anode, and a counter electrode, in our case the cathode (Fig. 1) (Helm et al., 2010). The operation mode is potentiostatic, which means that a constant potential is applied between the reference and the working electrode. When a chemical substance reacts at the working electrode, e.g. nitrite is oxidized at the anode, electrons are transferred between the substance and the working electrode. When, at the same time, a chemical substance reacts at the counter electrode, e.g. protons are reduced to hydrogen at the cathode, an electric current is generated between the working and counter electrode (Hamann and Vielstich, 2005). This current can be set in correlation to the concentration of the analyte, i.e. the substance, which reacts at the working electrode. In contrast to the potentiometric measuring principle, which is based on a passive registration of the potential difference between two electrodes (Schwedt et al., 2016) and is applied in ion-selective electrode (ISE) sensors for ammonium or nitrate, amperometric measurement is based on actively controlling the potential at the working electrode and measuring the resulting electric current. The amperometric measuring principle has been studied and commercialized and normally requires a membrane or some other diffusion barrier (Helm et al., 2010) in order to achieve selectivity. Amperometric sensors are available e.g. for dissolved oxygen (Clark electrode) (Hamann and Vielstich, 2005), ozone (e.g. 9185 sc Ozone amperometric analyzer, Hach Lange GmbH, Rheineck, Switzerland) (Helm et al., 2010) and free chlorine (e.g. analog free chlorine sensor CCS51, Endress + Hauser, Reinach, Switzerland).

According to the review of Helm et al. (2010), temperature influences amperometric measurements since it affects the rate of diffusion. With temperature, the width of the diffusion layer decreases and the diffusion coefficient increases, which causes a higher electric current (Ahmad, 2006). Furthermore, a low pH could cause a higher signal because more protons are available for reduction at the cathode. We also expect an effect of the electric conductivity in the electrolyte since it facilitates the exchange of electrons in the liquid.

Mixing conditions could also have an impact on the sensor signal, because they influence the thickness of the diffusion layer (Helm et al., 2010). Furthermore, the oxygen concentration could influence the amperometric measurement since oxygen has a high redox potential and is easily reduced with protons to water at the cathode. Air bubbles could also affect the measurement since they pose both electrochemical resistance and mass transfer barriers to the electrode reactions (Zhang and Zeng, 2012). The aeration rate in the nitrification reactor combines these effects.

The aim of this study was to evaluate whether an amperometric sensor allows a reliable in-situ measurement of nitrite, which could later be used for process control of urine nitrification. For this purpose we determined the required upper limit of the working range of a nitrite sensor for urine nitrification. Furthermore, we studied the robustness of the sensor signal against changes in the operational conditions such as temperature, pH, electric conductivity and aeration rate. We also assessed the effects of typical wear-and-tear of the amperometric nitrite sensor, i.e. we investigated the drift behaviour as well as fouling, which is one important root cause of drift. The overall hypothesis of our study was that an amperometric sensor is a robust and simple method to monitor nitrite concentrations during urine nitrification.

## 2 Materials and methods

In the following chapters we describe the amperometric nitrite measurement principle and the experimental design we used to test its hardware implementation. Furthermore, we explain how we assessed the necessary upper limit of the working range for urine nitrification and how the collected data were analysed. Finally, we give an overview about the conducted experiments.

### 2.1 Amperometric nitrite measurement

In our study of the amperometric sensor, the nitrified urine was the electrolyte and nitrite the analyte. We chose graphite for the working and counter electrode because it is rather cheap and has been applied in urine treatment before. A potentiostat (PGU 10V-IMP-S, IPS Elektroniklabor GmbH & co. KG, Germany) and the software EcmWin (V2.4) were used to hold the required potential between the reference and working electrode as well as to measure the occurring electric current. In amperometric nitrite measurement, the target reaction is the selective nitrite oxidation to nitrate that occurs at the anode, which is the working electrode. Previous experiments in our lab showed that an anode potential of 1.20 V vs. standard hydrogen electrode (SHE) is suitable for a selective nitrite oxidation at the anode. Fig. 1 shows a scheme of the measurement setup.

**Fig. 2:**
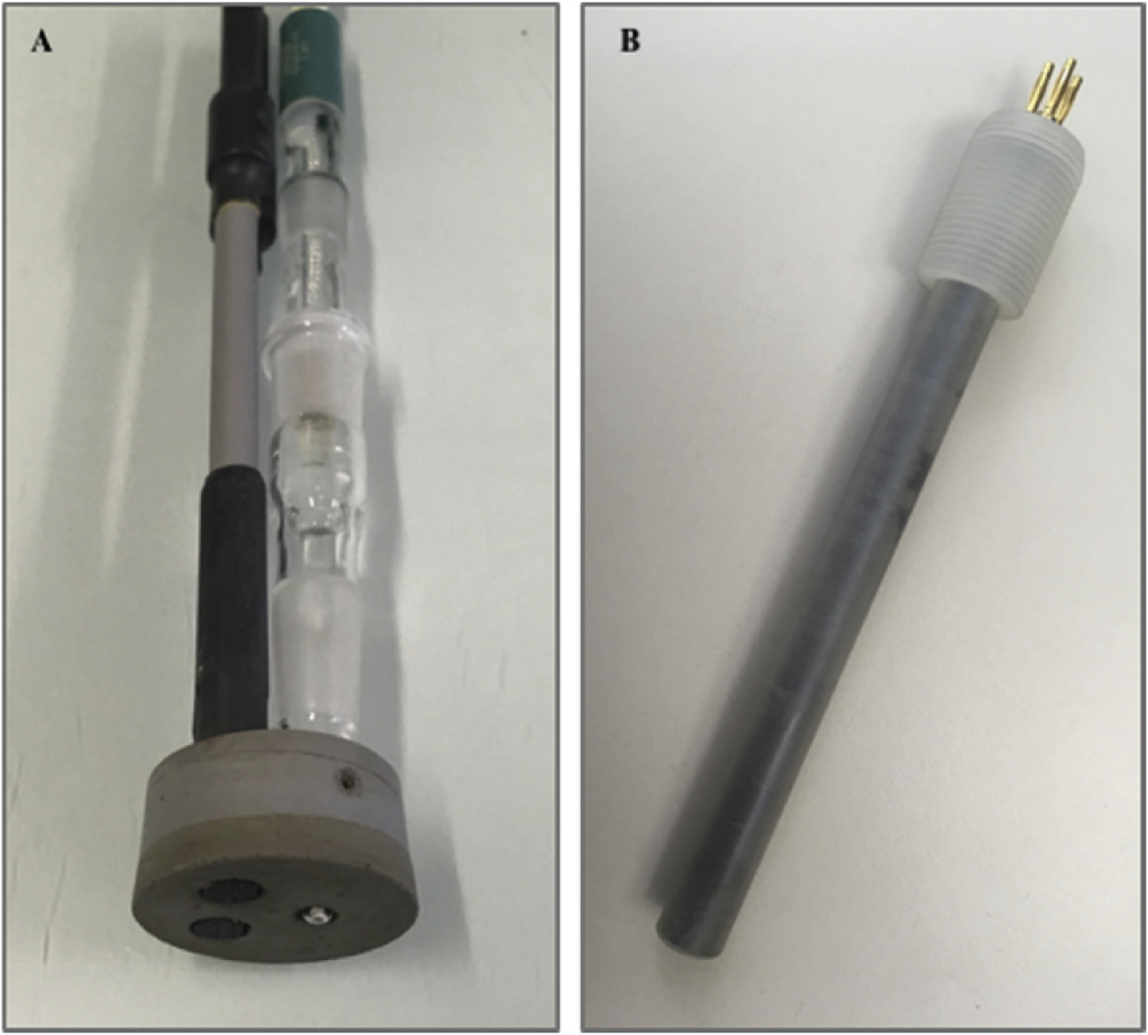
Picture of the large (A) and the small (B) amperometric nitrite sensor.

We studied two variants of the amperometric nitrite sensor both of which were not equipped with a membrane or another diffusion barrier. Firstly, a large sensor was tested which is a handmade prototype (Fig. 2A): Two graphite electrodes (isostatically fine pressed graphite, Ø = 7 mm, R 8650, SGL Carbon GmbH, Germany) served as working and counter electrode. A silver-chloride reference electrode was used (Ag/AgCl, E = +0.21 V vs. SHE, In-Lab ® reference, in 3 M KCl, Mettler-Toledo, Switzerland) and was mounted into a Luggin capillary filled with 3 M KCl solution. The Luggin capillary and the graphite electrodes were put into a rubber cone and inserted into a PVC-pipe. The electrodes were placed in a way that they did not protrude from the rubber cone and had a reactive surface of 38 mm^2^, each. The potential between the reference and the working electrode was set to a value of E = 0.99 V vs. Ag/AgCl_2_, which corresponds to 1.20 V vs. SHE.

The second variant of the sensor was smaller and manufactured by IPS Elektroniklabor GmbH & Co. KG, Germany (Fig. 2B). Three graphite electrodes were used (Ø = 3 mm, R 8650, TVB GmbH, Germany): two of them served as working and counter electrode, respectively, and the third electrode was the reference electrode. Experiments in our lab resulted in an approximate standard electrode potential of graphite as a reference electrode in nitrified urine of 0.31 V vs. SHE. The electrodes were sealed with a highly isolating material (PURe Isolation ST 33, copaltec GmbH, Germany) within the housing (Ø = 12 mm). The electrodes did not protrude and had a reactive surface of 7 mm^2^, each. The potential between the reference and the working electrode was set to a value of 0.89 V vs. graphite, which approximately corresponds to 1.20 V vs. SHE.

### 2.2 Experimental setup

We tested the amperometric nitrite sensor in a continuous stirred-tank reactor (CSTR) for urine nitrification at Eawag in Dübendorf. The liquid volume was between 120 L and 140 L and the reactor was operated with a two-point control based on pH as was implemented by Udert and Wächter (2012). The average total ammonia concentration of the urine that was fed to the reactor was 2870 ± 330 mg_N_/L and the pH was 8.8 ± 0.1. Further details to the operation of the CSTR can be found in Fumasoli et al. (2016).

### 2.3 Determination of the required upper limit of the working range

In order to define the upper limit of the nitrite range to be covered by the sensor, we determined the optimal nitrite concentration for urine nitrification. According to Anthonisen et al. (1976), NOB use NO_2_^−^ as substrate but are inhibited by HNO_2_. As a consequence, the optimal nitrite concentration for NOB activity depends on the pH value the NOB are exposed to. Based on the model of Fumasoli (2016) and constants derived by Reimann (2019), we derived an equation for NOB activity (Equation (1)), determined its maximum and solved it for the nitrite concentration [NO_2_^−^]_opt_ (Equation (2)). We defined [HNO_2_] according to the acidic equilibrium in dependency of the nitrite activity, pH and the dissociation constant for nitrite. The ionic strength was estimated to be 0.16 mol/L based on the concentration of the major inorganic ions. By using this ionic strength and the Davies approach (Stumm and Morgan, 1996) we calculated an average activity coefficient of 0.75 for single charged ions. The detailed explanation of this assessment can be found in the Supplementary Material S1.1.1.

(1) $$\frac{dX_{\mathrm{NOB}}}{\mathrm{dt}}=\mu_{\max}*\frac{K_{I,\mathrm{HN}O_{2}}}{\left[\mathrm{HN}O_{2}\right]+K_{I,\mathrm{HN}O_{2}}}*\frac{\left[NO_{2}^{-}\right]}{\left[NO_{2}^{-}\right]+K_{S,NO_{2}^{-}}}*\frac{K_{I,NH_{3}}}{\left[NH_{3}\right]+K_{I,NH_{3}}}*X_{\mathrm{NOB}}$$

(2) $${\left[NO_{2}^{-}\right]}_{\mathrm{opt}}=\;\sqrt{\frac{K_{I,\mathrm{HN}O_{2}}*K_{S,NO_{2}^{-}}*K_{NO_{2}^{-}}*10^{\mathrm{pH}}}{f_{A,NO_{2}^{-}}}}$$

$\frac{dX_{NOB}}{dt}=$ NOB growth rate $\left[g_{COD}/\left(L*d\right)\right]$
$\mu_{max}=$ maximum growth rate [1/d]
$K_{I,HNO_{2}}=$ constant for non-competitive inhibition by HNO_2_ (= 0.138 (Reimann, 2019)) $\left[mg_{N}/L\right]$
$K_{S,NO_{2}^{-}}=$ affinity constant for NO_2_^−^ (= 1.46 (Reimann, 2019)) $\left[mg_{N}/L\right]$
$K_{I,NH_{3}}=$ constant for non-competitive inhibition by NH_3_ (= 72.5 (Reimann, 2019)) $\left[mg_{N}/L\right]$
$X_{NOB}=$ biomass concentration of NOB $\left[g_{COD}/L\right]$
$K_{NO_{2}^{-}}=$ acid dissociation constant for NO_2_^−^ (= 10^−3.29^ at 25 °C (Schwartz and White, 1981)) [−]
$f_{A,NO_{2}^{-}}=$ activity coefficient for NO_2_^−^ (= 0.75) [−]

Besides the theoretical calculations, we estimated the nitrite concentration for maximum activity of NOB based on results of Thürlimann et al. (2019). According to their study, the course of the nitrite concentrations during nitrite degradation by NOB shows an inflection point, which corresponds to the value where the NOB are most active. At this point the kinetics of NOB change from substrate inhibition to substrate limitation. Based on this approach we determined the optimal nitrite concentration at pH values between 5.9 and 6.1. Details can be found in the Supplementary Material S1.1.2.

### 2.4 Analytical methods

#### 2.4.1 Signal processing

The measurement frequency for both nitrite sensors was 1 Hz. The data were smoothened with the median of 30 s for all in-situ experiments. For the evaluation of the response time of the amperometric nitrite sensor (see Chapter 2.5.4) the sensor signal was not smoothened but was used in its raw form. The sensor signal measured during the signal trend experiment (see Chapter 2.5.6) was smoothened with a 5 min moving median. For the other ex-situ experiments, the median of 2.5 min was taken for each data point starting 30 s after the sensor was put into the stock solution in order to minimize initial response time effects.

#### 2.4.2 Determining the calibration curve

For each month, we took all data points which lay between 0 mg_N_/L and the upper limit of the working range and fitted a linear curve with a least squares fit. The standard deviation of the method s_xo_ was calculated according to ISO (1990). The lower s_xo_ the better the performance of the analytical method. Furthermore, we calculated the coefficient of determination R^2^ which is a statistical measure of how well the model represents the real measurements (Fahrmeir et al., 2016). An R^2^ of 1 means that the model perfectly fits the data points.

#### 2.4.3 Model structure identification

We used a polynomial regression model describing the sensor signal, i.e. the current density, as a function of the variables nitrite, temperature and pH. Polynomial terms up to a power of 3 were tested, leading to a total of 10 terms for the most complex model (Equation (3)). In order to test which terms improve the model, we created all possible combinations of these terms by setting some of the parameters to zero. This resulted in 2^10^ = 1024 different models which were compared according to their root mean square error (RMSE). We compared the most complex function that includes all terms (Equation (3)) to the linear model only including the nitrite concentration (Equation (4)) in order to determine whether the inclusion of temperature and pH improved the estimation of the electric current. In the Supplementary Material S1.2, the model identification procedure is explained in more detail.

(3) $$j=\beta_{0}+\beta_{\left[NO_{2}^{-}\right]}*\left[NO_{2}^{-}\right]+\beta_{{\left[NO_{2}^{-}\right]}^{2}}*{\left[NO_{2}^{-}\right]}^{2}+\beta_{{\left[NO_{2}^{-}\right]}^{3}}*{\left[NO_{2}^{-}\right]}^{3}+\beta_{T}*T+\beta_{T^{2}}*T^{2}+\beta_{T^{3}}*T^{3}+\beta_{pH}*pH+\beta_{pH^{2}}*pH^{2}+\beta_{pH^{3}}*pH^{3}$$

(4) $$j=\beta_{0}+\beta_{\left[NO_{2}^{-}\right]}*\left[NO_{2}^{-}\right]$$

#### 2.4.4 Chemical analysis

Previous to chemical analysis the grab samples were filtered through 0.4 μm filter papers (MN GF-5, MACHERY-NAGEL GmbH & Co. KG, Düren, Germany). The nitrite concentrations were measured with spectrophotometric cuvette tests (LCK 341 with a measurement range of 0.015 – 0.6 mg_N_/L and LCK 342 with a measurement range of 0.6 – 6 mg_N_/L, Hach Lange GmbH, Germany) using a spectrophotometer from Hach Lange GmbH (DR 2800, Hach Lange GmbH, Germany). With these cuvette tests, we typically achieve a measurement accuracy of 1%.

### 2.5 Experiments

#### 2.5.1 Temperature and pH

In order to test the sensor’s dependency on temperature and pH, 79 random samples from the urine nitrification reactor within the aimed nitrite range at temperatures between 22.5 and 26.5 °C and at pH values between 6.0 and 7.1 were taken and compared with current density measurements with the large sensor (Supplementary Material S1.3). The temperature variability resulted from the exhaust heat of a distiller, that was placed in the room of the experimental setup, and was not controlled. In order to achieve different pH values the pH set point was adapted in the nitrification control.

#### 2.5.2 Aeration

Another goal of this study was to assess which influence different aeration rates have on the amperometric nitrite measurement. For this purpose, we collected 58 random samples at aeration rates between 0 and 0.6 Nm^3^/h with the large sensor from the urine nitrification reactor. In order to receive different nitrite concentrations for every aeration rate, the pH set point was increased or decreased during each test.

#### 2.5.3 Electric conductivity

We tested the influence of electric conductivity also. We conducted an ex-situ experiment with stock solutions at nitrite concentrations of 25 mg_N_/L and 50 mg_N_/L (NaNO_2_, assay = 99%, Merck KGaA, Darmstadt, Germany) and at different levels of conductivity. The electric conductivity was adjusted with sodium chloride (assay ≥ 99.5%, Merck KGaA, Darmstadt, Germany) to temperature corrected values of 10, 20, 30, 40 and 50 mS/cm. We further produced stock solutions at 25 mg_N_/L and 50 mg_N_/L without adding sodium chloride, which resulted in electric conductivities of 0.3 mS/cm and 0.5 mS/cm, respectively. 250 mL of each stock solution were mixed with a magnetic stirrer (color squid white, IKA®, Staufen, Germany) at 400 rpm and the current density was measured with the small sensor. The measurements were repeated 4 times.

#### 2.5.4 Response time

The assessment of the response time was executed according to ISO 15839 (ISO, 2003). In this initial experiment, we considered the working range to be 0 to 24 mg_N_/L. Therefore, the response time assessed in this study is only valid for that range. Details to the procedure and calculations can be found in the Supplementary Material S1.4.

#### 2.5.5 Typical wear-and-tear

In order to evaluate whether the signals produced by the nitrite sensor exhibit drift, and when so, whether the root cause of drift includes fouling, in addition to corrosion or disintegration of the sensor, we conducted ex-situ validation tests. Stock solutions of nitrite concentrations between 2 and 34 mg_N_/L were prepared using NaNO_2_ (assay = 99%, Merck KGaA, Darmstadt, Germany) and nanopure water. No additional salt was added to these solutions. 250 mL of the stock solutions were put into a glass beaker and continuously stirred with a magnetic stirrer at 400 rpm (color squid white, IKA®, Staufen, Germany). The current density was measured for each concentration before and after cleaning the sensor and a linear curve of the current density in dependency of the nitrite concentration was fitted with a least squares fit. First, the ex-situ validation of the sensor was executed weekly, then every two weeks and the last measurement was executed after the sensor was in the nitrification reactor for 5.5 weeks. The ex-situ experiments were conducted with the large sensor.

The fouling effect on the small sensor was tested with nitrified urine in a similar ex-situ experiment. Nitrified urine without detectable nitrite was collected and spiked with NaN_3_ (assay ≥ 99%, Merck Schuchardt OHG, Hohenbrunn, Germany) in order to stop biological activity. Six solutions were produced at nitrite concentrations of 0, 10, 20, 30, 40 and 50 mg_N_/L (NaNO_2_, assay = 99%, Merck KGaA, Darmstadt, Germany). 250 mL of the stock solutions were stirred with a magnetic stirrer at 400 rpm (color squid white, IKA®, Staufen, Germany). The experiment was conducted after the sensor had been in the nitrification reactor for 10 days. The current density in each of these solutions was measured before and after cleaning the sensor. A linear curve of the current density as a function of the nitrite concentration was fitted with a least squares fit for the data before and after cleaning the sensor.

#### 2.5.6 Ex-situ versus in-situ

To determine whether the nitrite sensor can be calibrated ex-situ, we compared the calibration curves obtained from an ex-situ experiment with the small sensor with synthetic nitrite solutions at 0, 5, 10, 15, 20, 25, 35, 40, 45 and 50 mg_N_/L (NaNO_2_, assay = 99%, Merck KGaA, Darmstadt, Germany) and an electric conductivity of 16 mS/cm (temperature corrected, NaCl, assay ≥ 99.5%, Merck KGaA, Darmstadt, Germany) and from the in-situ measurements. The in-situ data were collected in November 2018 with the small sensor. This experiment was not conducted with the large sensor.

Furthermore, the signal trend of a synthetic nitrite solution at a nitrite concentration of 25 mg_N_/L (NaNO_2_, assay = 99%, Merck KGaA, Darmstadt, Germany) and an electric conductivity of 16 mS/cm (NaCl, assay ≥ 99.5%, Merck KGaA, Darmstadt, Germany) was observed with the small sensor over 400 min while it was stirred with a magnetic stirrer at 400 rpm (color squid white, IKA®, Staufen, Germany). The pH and the dissolved oxygen concentration were measured and logged (pH 340 and Cond 340i, WTW, Weilheim, Germany) in order to be able to investigate the influence of these parameters.

## 3 Results

### 3.1 Determination of the required upper limit of the working range

**Fig. 3:**
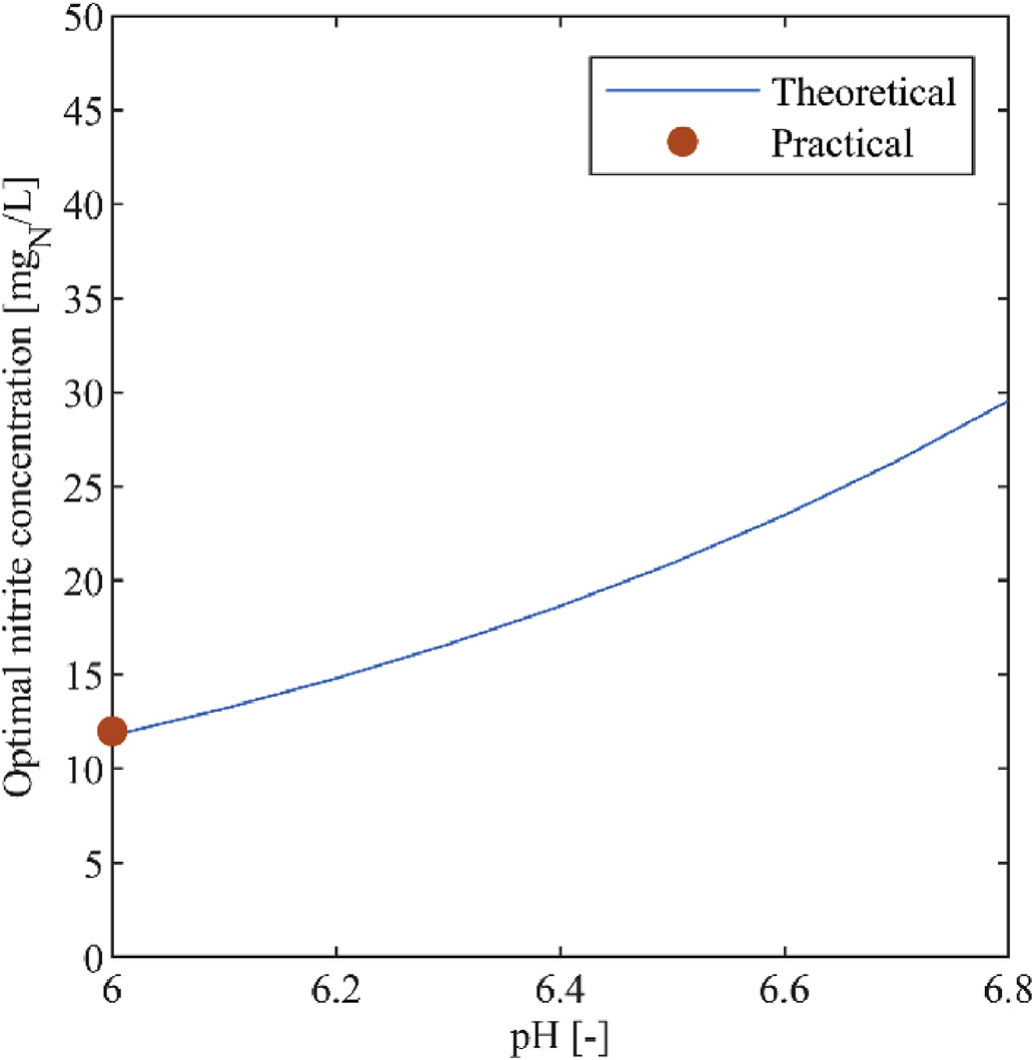
The optimal nitrite concentration as a function of pH based on the theoretical and practical approach. The theoretical calculations were done with an average ionic strength of 0.16 mol/L and an average activity coefficient of 0.75 for a temperature of 25 °C.

Our calculations resulted in optimal nitrite concentrations [NO_2_^−^]_opt_ of 12–30 mg_N_/L at a pH between 6.0 and 6.8 (Fig. 3). These values are based on Equation (2), using an average ionic strength of 0.16 mol/L and an average activity coefficient for single charged ions of 0.75.

According to Thürlimann et al. (2019), the inflection point in the downward trend of the nitrite concentration corresponds to the point of maximal NOB activity. Using the data of Thürlimann et al. (2019), we estimated that this inflection point occurs at 12 mg_N_/L at a pH between 5.9 and 6.1, which confirms the theoretical calculations shown in Fig. 3.

To ensure maximum NOB performance and prevent inhibition by nitrite the urine nitrification reactor should be operated close to [NO_2_^−^]_opt_. According to ISO 8466-1 (ISO, 1990), the most frequently expected concentration should lie in the centre of the working range of an analytical method. Since we expect the optimal nitrite concentration to be in a range of 12–30 mg_N_/L, the upper limit of the working range should be between 24 and 60 mg_N_/L. Our subjective yet informed decision is that the sensor should be able to reliably measure concentrations up to 50 mg_N_/L.

### 3.2 Data calibration

The linear calibration curves of all months resulted in a standard deviation of the method s_xo_ of 4 mg_N_/L or less (Table 1). The measurements from July 2018 resulted in a particularly low standard deviation because the four samples from that month were collected during one day only, so that environmental conditions or sensor properties were very similar for all four measurements. The coefficients of determination R^2^ show that, overall, quadratic curves did not result in substantially better fits than linear curves. More details to the calibration curves can be found in the Supplementary Material S2.1.

**Table 1:** Number of samples and their mean, standard deviation of the method s_xo_ and coefficient of determination R^2^ of the monthly separated data from the large and small sensor. The temperature was never controlled but natural fluctuations of the room temperature occurred.

	Operating conditions	Number of samples	Mean [mg_N_/L]	s_xo_ linear [mg_N_/L]	R^2^ linear [−]	R^2^ quadratic [−]
Large sensor						
April 2018	pH variations	42	20 ± 16	3.0	0.964	0.974
May 2018	pH variations	37	25 ± 15	3.4	0.952	0.966
June 2018	Aeration rate variations	54	25 ± 15	3.2	0.955	0.954
July 2018	–	4	26 ± 21	1.0	0.999	0.999
Small sensor						
October 2018	pH variations	19	21 ± 15	2.7	0.970	0.978
November 2018	pH and aeration rate variations	14	23 ± 15	2.3	0.977	0.975
January 2019	pH and aeration rate variations	17	23 ± 17	4.0	0.948	0.946
February 2019	–	3	21 ± 20	2.4	0.993	1.000

**Fig. 4:**
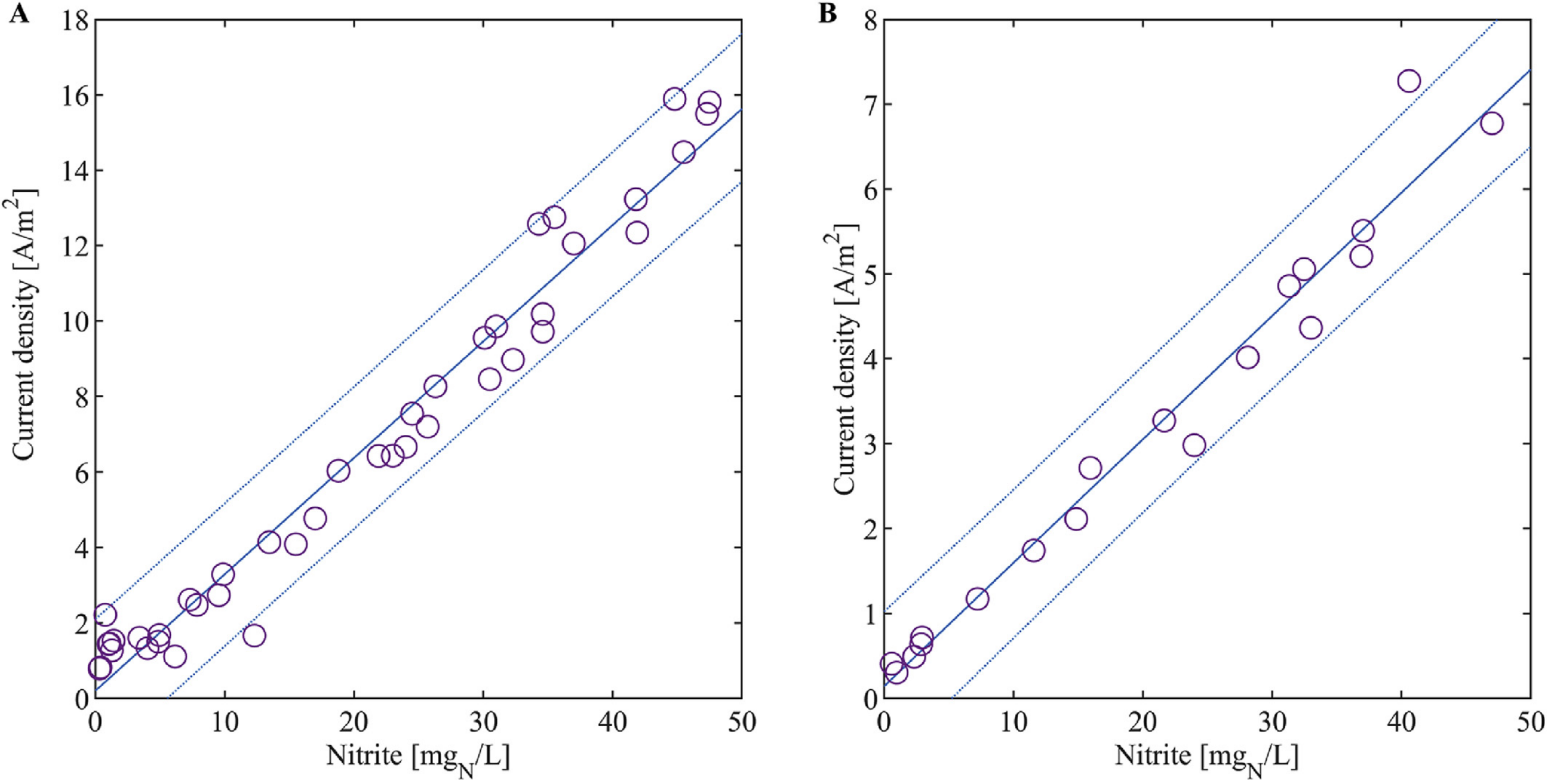
Data in the nitrite range of 0 – 50 mg_N_/L with the linear fit and the corresponding 95%-confidence interval. (A) Data from the nitrification reactor during April 2018 with the large sensor. (B) Data from the nitrification reactor during October 2018 with the small sensor.

As an example for a data fit, Fig. 4 shows the measurements with the large sensor in April 2018 and with the small sensor in October 2018 with a linear fit and the 95%-confidence interval. It can be seen that the offsets of the functions are close to 0 A/m^2^ and the 95%-confidence interval covers 0 A/m^2^. Since all fits showed similar small offsets and linear behaviour, we assume that a linear curve through the origin is suitable for calibration. As a consequence, for practical applications a one-point calibration of the sensitivity only might be sufficient. However, in our investigations, we determined both the offset and sensitivity to obtain maximum accuracy..

**Fig. 5:**
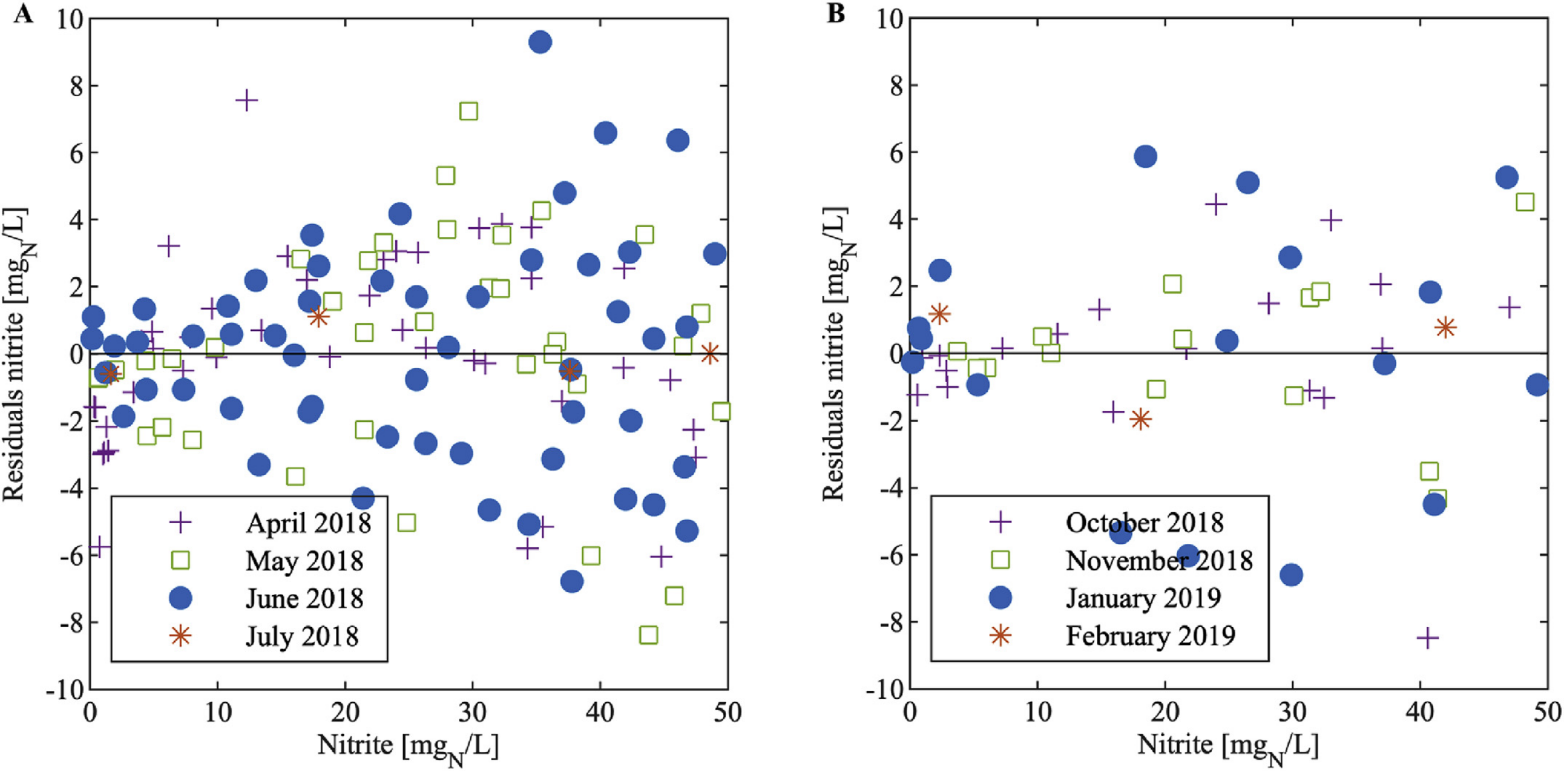
Residuals of the nitrite concentration of the large (A) and the small (B) sensor for each month.

**Fig. 6:**
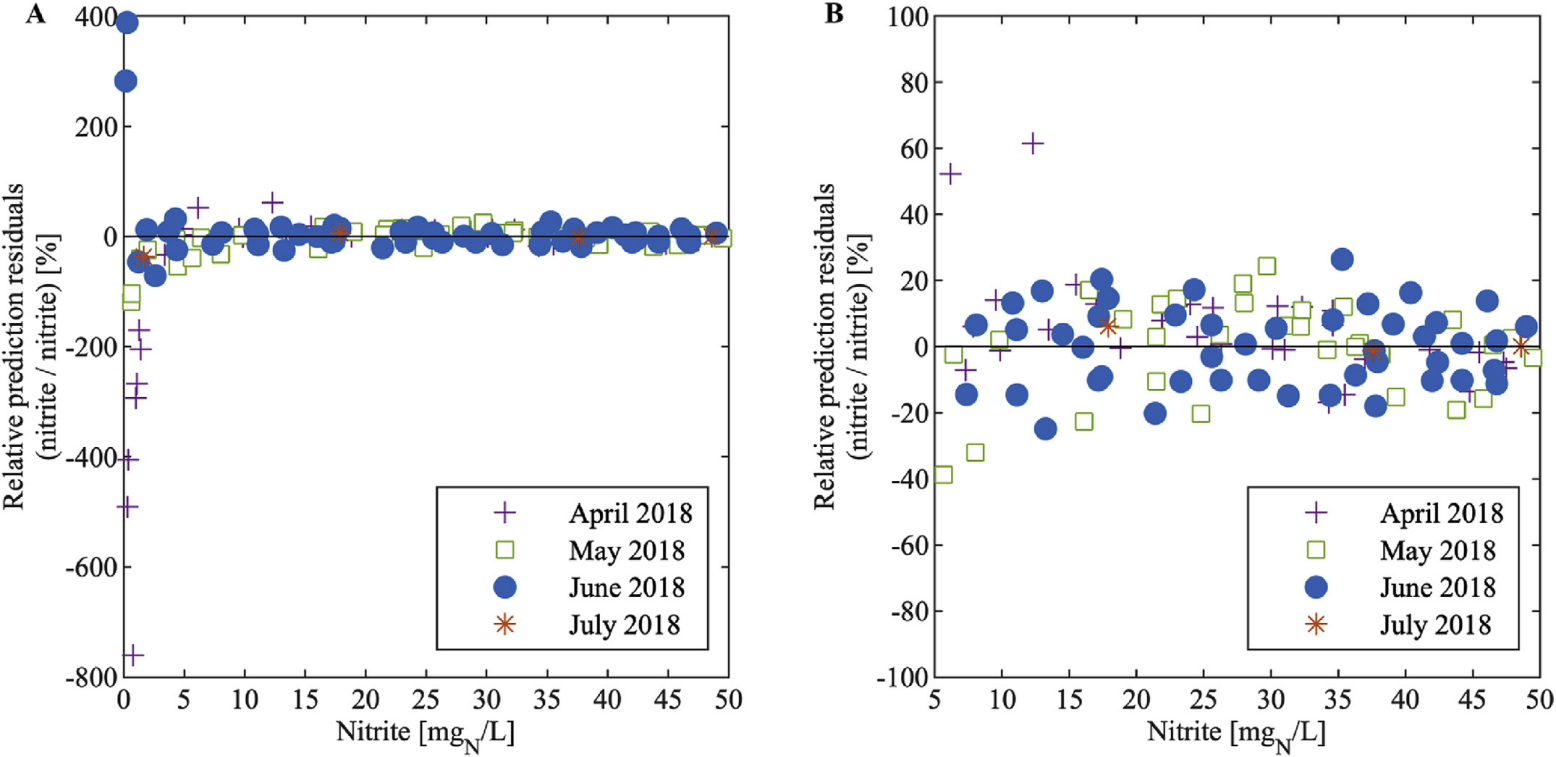
Relative prediction residuals of the nitrite concentration in dependency of the nitrite concentration of the large sensor for each month showing the range of (A) 0–50 mg_N_/L and (B) 5 to 50 mg_N_/L.

**Fig. 7:**
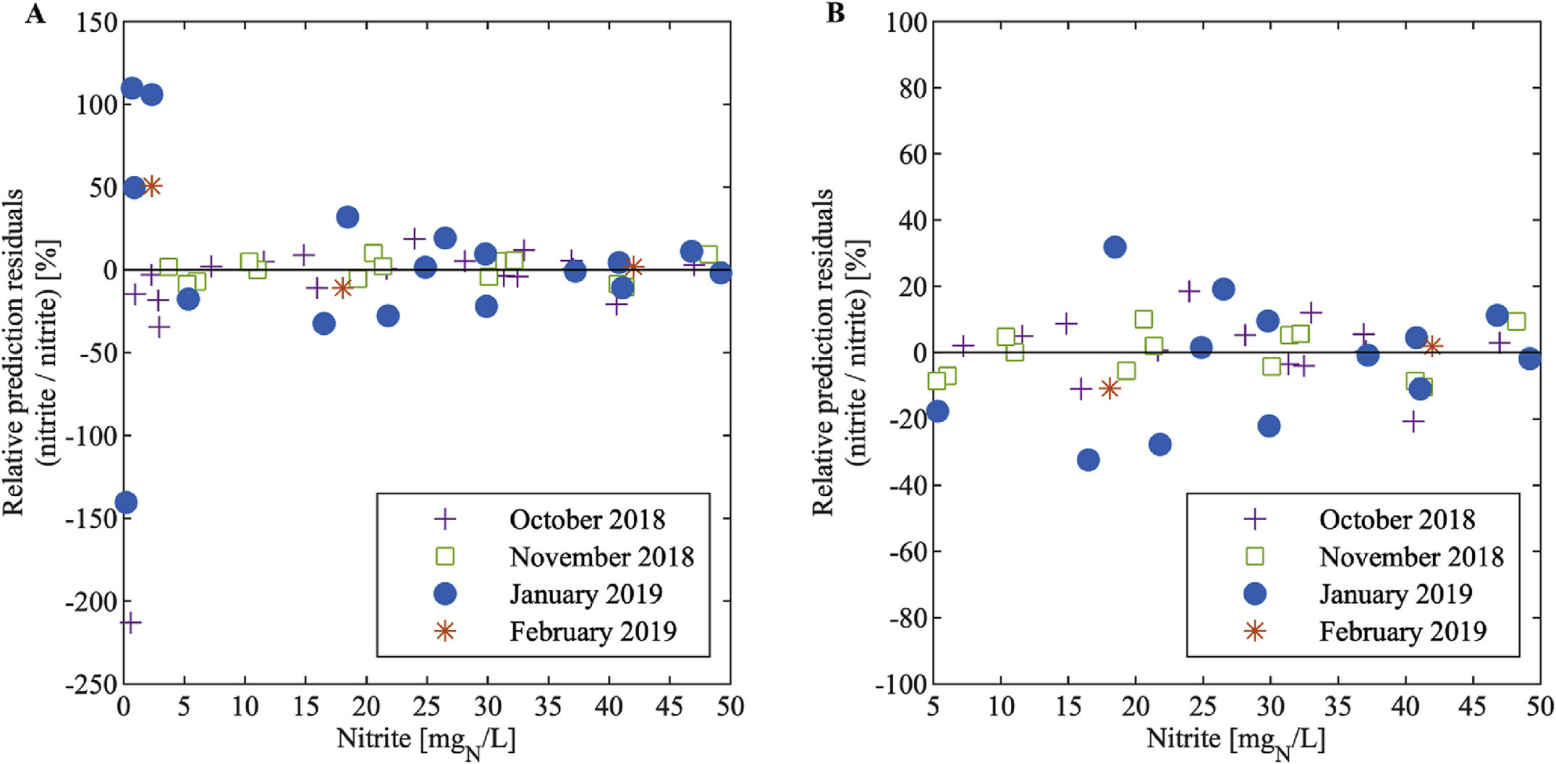
Relative prediction residuals of the nitrite concentration in dependency of the nitrite concentration of the small sensor for each month showing the range of (A) 0–50 mg_N_/L and (B) 5 to 50 mg_N_/L.

The spread of the nitrite concentration residuals generally increased with increasing nitrite concentrations (Fig. 5). However, the relative prediction residuals, that is the residuals after division by the nitrite concentration (Fig. 6 and Fig. 7), were nearly constant above a nitrite concentration of 5 mg_N_/L: 90% of the data above 5 mg_N_/L have a maximum relative deviation of 20% and 21% for the large and the small sensor, respectively. This means that the standard deviation of the measurement error is close to proportional to the measured value in this range. Below 5 mg_N_/L, the relative deviation increases substantially with decreasing nitrite concentration. Therefore, we conclude that measurements below 5 mg_N_/L have large errors and that the sample for the one-point calibration should be at a nitrite concentration higher than 5 mg_N_/L. We suggest to choose a point close to the centre of the working range since this is the concentration we expect most frequently, i.e. around 25 mg_N_/L (see Chapter 3.1).

### 3.3 Factors influencing the raw sensor signal

#### 3.3.1 Temperature and pH

The results obtained in April to May 2018 with the large sensor are discussed first. Experiments with the small sensor confirmed the signal’s dependencies on the nitrite concentration, temperature and pH. The data obtained with the large sensor are shown here, while the data obtained with the small sensor are shown in the Supplementary Material S2.2.

**Fig. 8:**
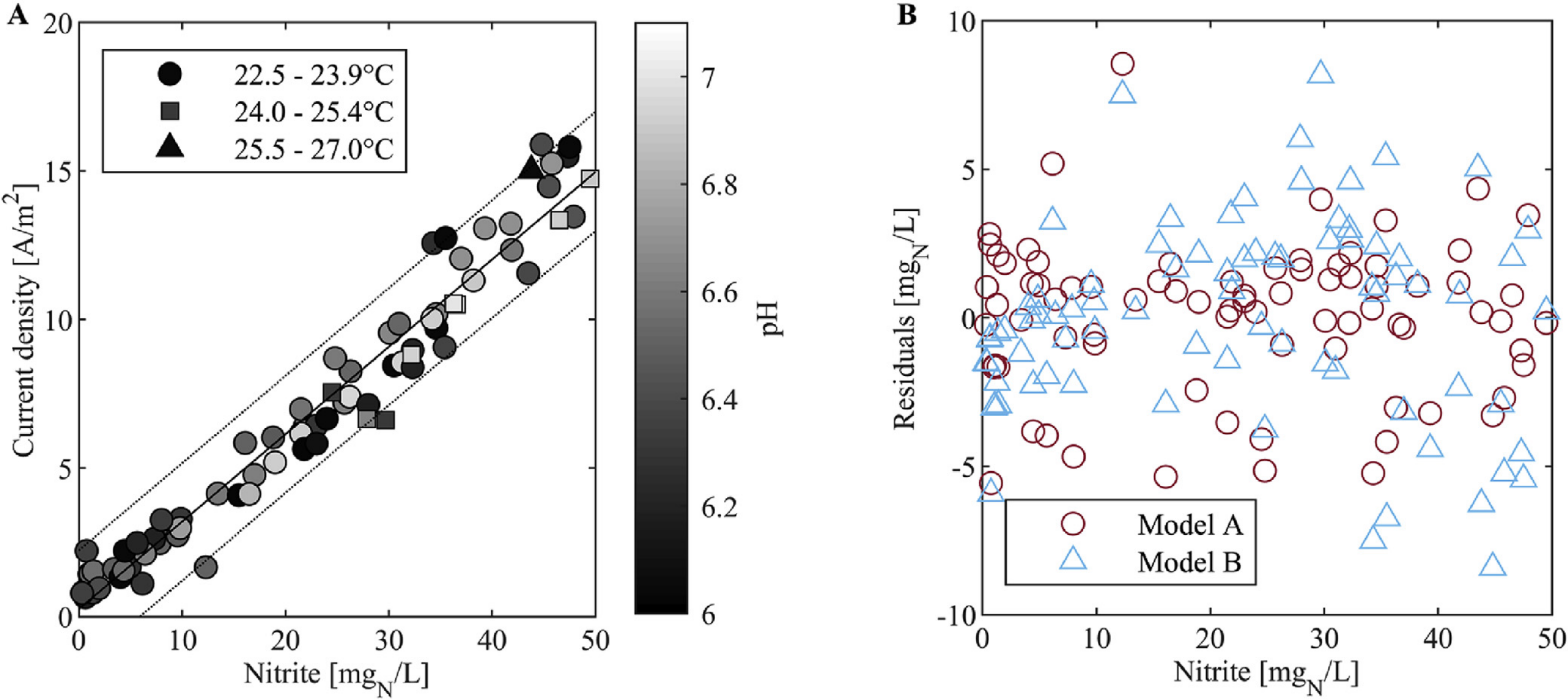
(A) Linear curve and the corresponding 95%-confidence interval describing the current density in dependency of nitrite, neglecting temperature and pH (Model B, Equation (4)). (B) Comparison of the residuals of the model best describing the correlation (Model A, Equation (3): including nitrite, temperature and pH) and the linear model only depending on nitrite (Model B). The results were obtained with the large sensor.

The polynomial regression showed that the current density can be described well when neglecting pH and temperature. The linear model (Model B, Equation (4): current density as a function of an offset and [NO_2_^−^], Fig. 8A) resulted in an RMSE of 1.0 A/m^2^. Model A, which best describes the data (Equation (3): current density as a function of an offset, [NO_2_^−^], [NO_2_^−^]^2^, [NO_2_^−^]^3^, T, T^2^, T^3^, pH, pH^2^, and pH^3^), resulted in an RMSE of 0.7 A/m^2^. Including temperature and pH increases the complexity of the model, yet does not offer a considerably lower RMSE than the linear model. Furthermore, when compared visually (Fig. 8B), it is apparent that the residuals of Model A and Model B lie in the same range. Therefore, we conclude that the influence of temperature and pH on the current density signal was negligible in our experiments. The standard deviation of the method s_xo_ of Model B was 3.3 mg_N_/L.

#### 3.3.2 Aeration

**Fig. 9:**
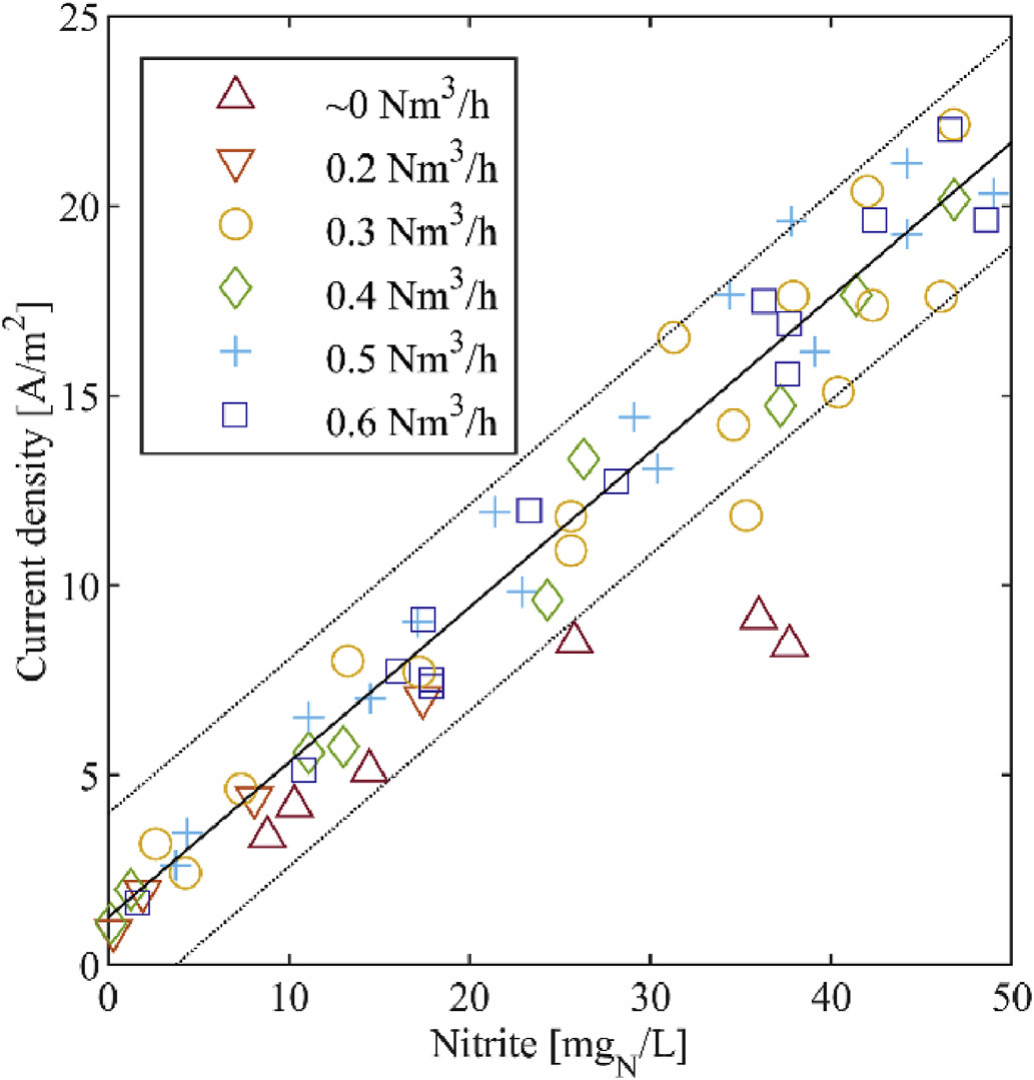
Samples of the aeration dependency assessment with the fitted linear curve (excluding the data at ∼0 Nm^3^/h) with the corresponding 95%-confidence interval. The results were obtained with the large sensor.

The assessment in June 2018 showed that the aeration rate does not influence the signal of the large sensor except at an aeration rate close to 0 Nm^3^/h (Fig. 9). When aeration was between 0 and 0.05 Nm^3^/h ± 3%, a lower signal was measured which we assume to be caused by diffusion limitation, i.e. a limitation of the transport of nitrite to the electrode due to poor mixing conditions. We merged the data from all assessed aeration rates, omitting the data at an aeration rate of approximately 0 Nm^3^/h. We fitted a linear curve to the current density in dependency of the nitrite concentration with a least squares fit. This resulted in a standard deviation of the method s_xo_ of 3.3 mg_N_/L. We conclude that the aeration rate does not influence the signal as long as the aeration rate in the reactor is 0.2 Nm^3^/h or more.

In the experiments with the small sensor we also did not find any influence of aeration on the measurement signal, when aeration was 0.2 Nm^3^/h or higher. The results can be found in the Supplementary Material S2.3.

#### 3.3.3 Electric conductivity

**Fig. 10:**
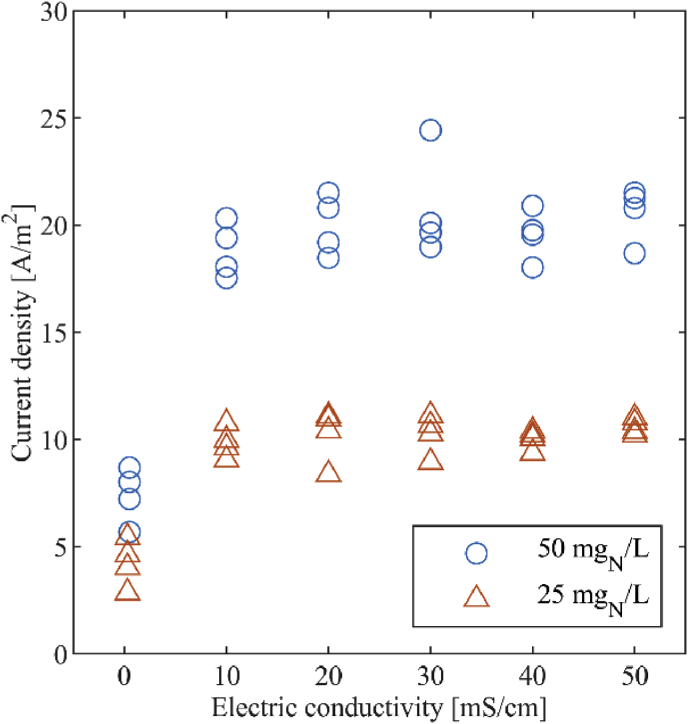
Current density measured at electric conductivities between 0 and 50 mS/cm, for artificial nitrite solutions at 25 mg_N_/L and 50 mg_N_/L. The results were obtained with the small sensor.

The assessment of the influence of electric conductivity showed that a difference in electric conductivity does not affect the sensor signal at 10 mS/cm or more (Fig. 10). However, as expected, at low electric conductivities of 0.3 mS/cm and 0.5 mS/cm, for 25 mg_N_/L and 50 mg_N_/L, respectively, we measured a lower current density. If the liquid to be measured is not conductive, an electrochemical measurement is not possible.

### 3.4 Response time

The assessment of the response time resulted in a rise time of 1.4 ± 0.8 s and a fall time below 5.2 ± 1.3 s. These results were obtained with synthetic nitrite solutions and response times in nitrified urine might be slightly different. Nevertheless, the experiment indicates that the sensor reacts within a few seconds to changes in the nitrite concentration.

### 3.5 Typical wear-and-tear

#### 3.5.1 Drift

**Fig. 11:**
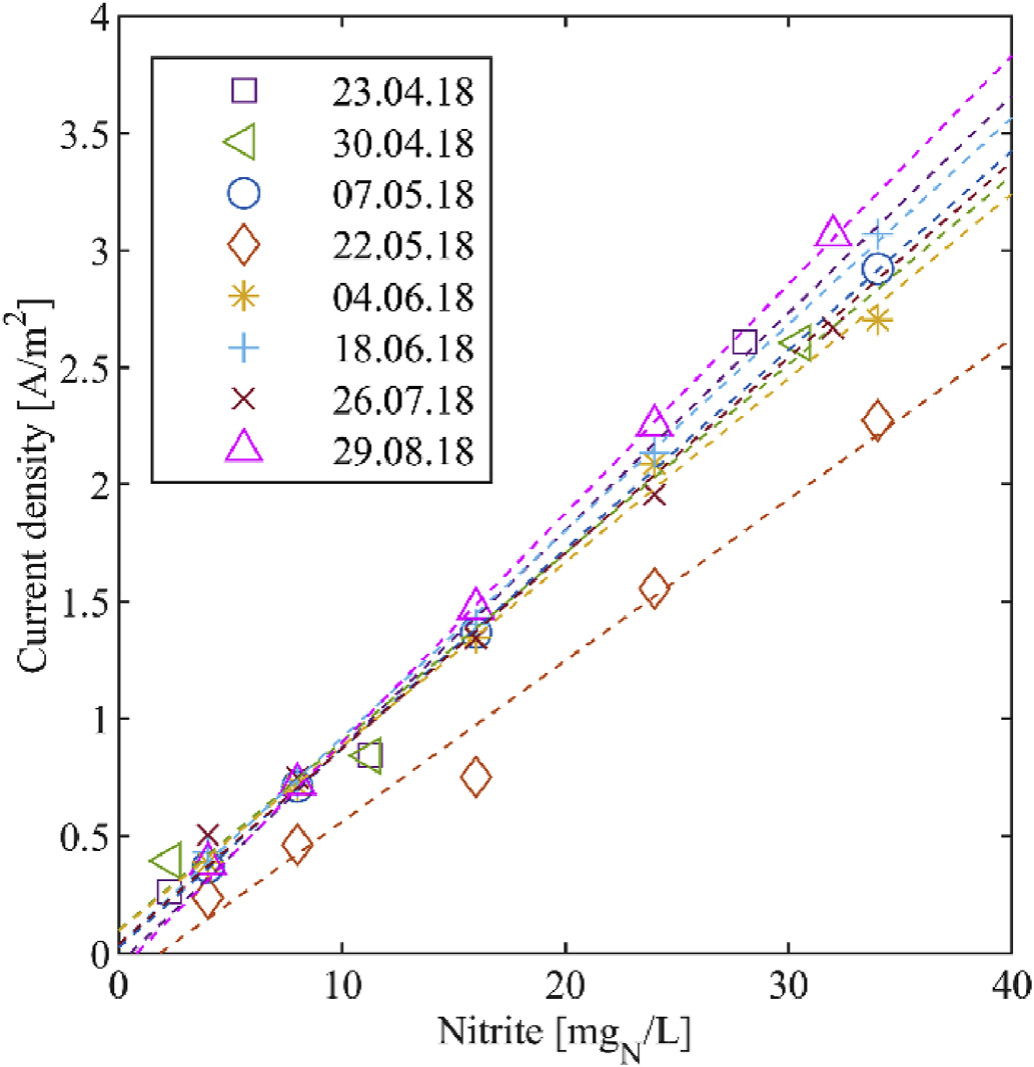
Results from the ex-situ drift analysis with the large sensor. The data points were collected after the sensor was cleaned.

In Fig. 11, one can view the calibration results obtained in the ex-situ experiments with the large sensor for a period of 4 months. The slopes of the fitted linear curves were 0.087 ± 8% (A/m^2^)/(mg_N_/L) and the offsets were in a range of −0.08 to 0.10 A/m^2^, with only one exception on May 22, 2018, thus suggesting a lack of drift for the offset as well as the sensitivity.

**Fig. 12:**
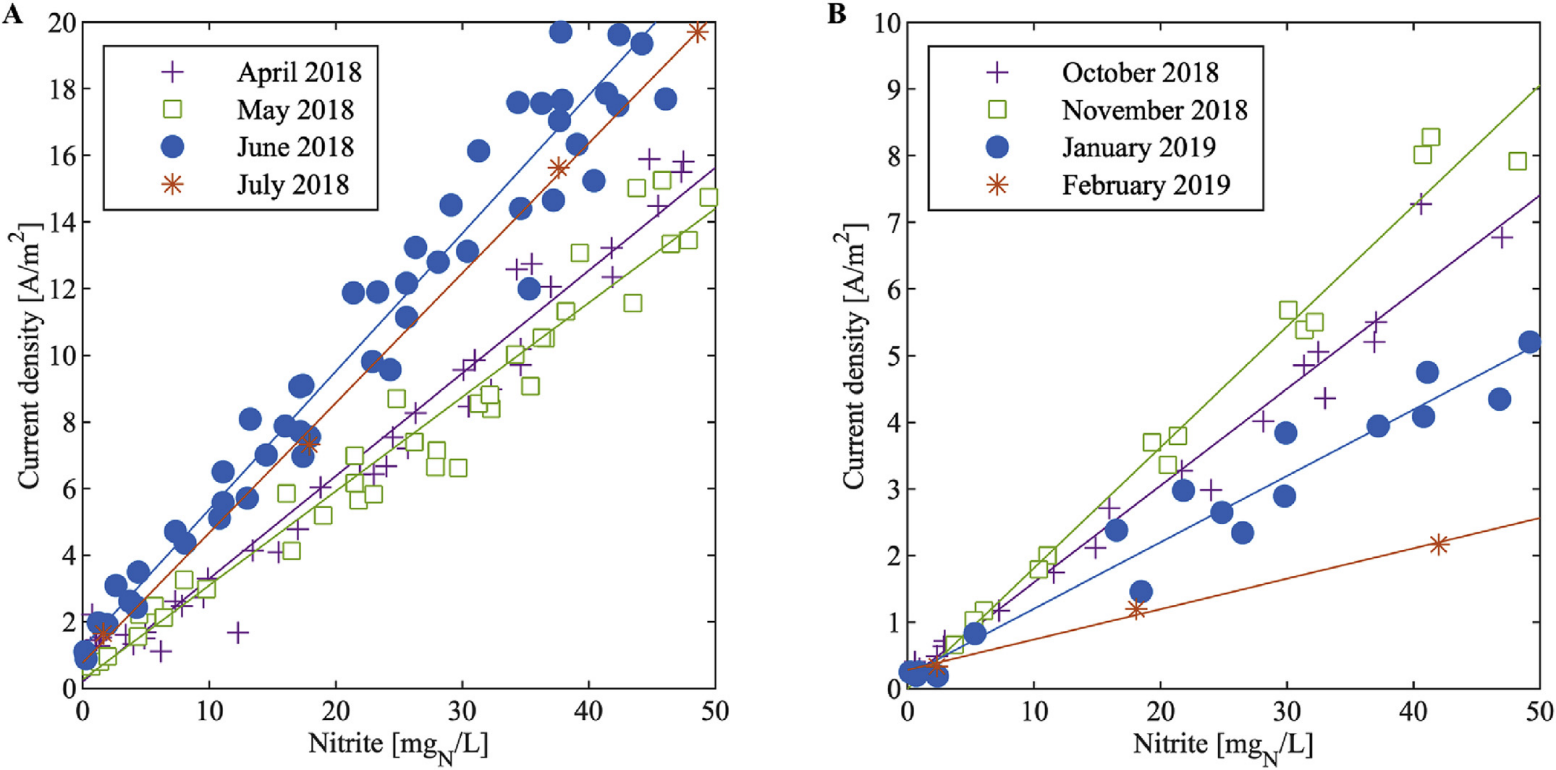
Results from the in-situ drift analysis with the large sensor (A) and the small sensor (B) over the nitrite concentration range of 0 – 50 mg_N_/L.

The in-situ measurements (Fig. 12A and B) also showed a rather stable offset close to 0 A/m^2^ (large sensor: 0.20–1.22 A/m^2^, small sensor: 0.00–0.29 A/m^2^) while the sensitivity of the calibration curve changed over time (large sensor: 0.349 ± 18% (A/m^2^)/(mg_N_/L), small sensor: 0.118 ± 61% (A/m^2^)/(mg_N_/L)). The sensitivity of the small sensor showed a stronger drift than the one of the large sensor, which might have two causes. First, the smaller reaction surface makes the sensor more susceptible to fouling or changes in the environmental conditions. Second, the reference electrode in the small sensor was graphite, while the large sensor had a conventional silver chloride reference. The graphite reference electrode might have been more prone to aging effects.

#### 3.5.2 Fouling

Control measurements did not show any significant fouling effects on the large sensor during its operation in the nitrification reactor, even after 5.5 weeks operation in the reactor. The ex-situ calibration curves, determined in synthetic nitrite solutions, show very similar slopes before and after cleaning (Supplementary Material S2.4).

In contrast, the ex-situ experiment with the small sensor, which was executed with nitrified urine, showed a stronger effect of fouling (Supplementary Material S2.4). The offsets lie close together while the sensitivity of the calibration curve is different before and after cleaning the sensor. As expected, the signal is higher after cleaning since there is no biofilm that either consumes the nitrite before it is measured or limits nitrite diffusion.

The different outcome of these experiments might be due to differences in the sensor construction. However, we assume that the different results were caused by the medium composition, which varies in factors such as particles, background matrix, electric conductivity or density.

### 3.6 Ex-situ versus in-situ

**Fig. 13:**
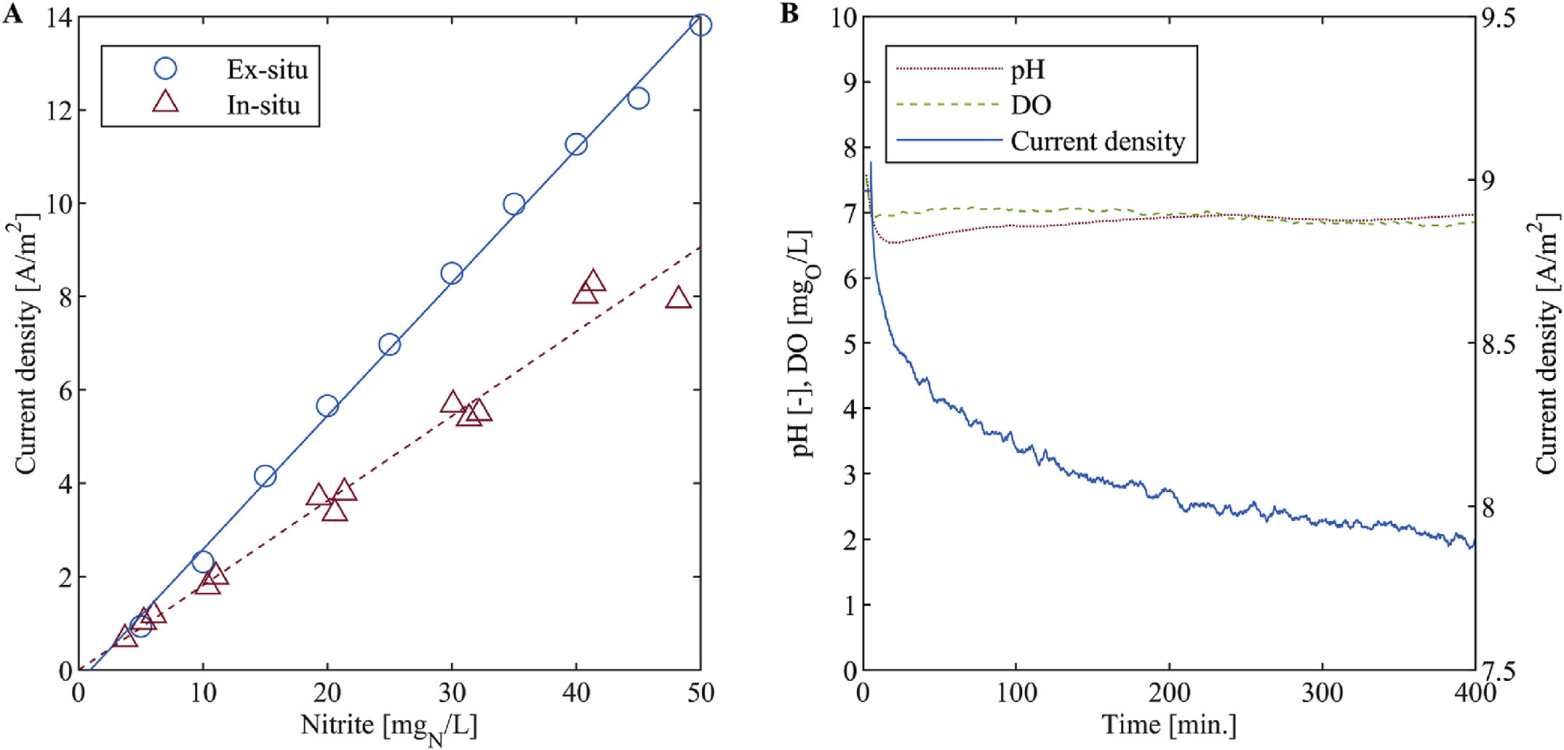
**(A)** Comparison of the ex-situ and in-situ calibration curves from November 2018. **(B)** Trend of the sensor signal of an artificial nitrite solution at 25 mg_N_/L, the pH and the dissolved oxygen concentration. The data were collected with the small sensor.

We found that the amperometric nitrite sensor should be calibrated in-situ rather than ex-situ. The small sensor measured a higher current density ex-situ (synthetic nitrite solution with an electric conductivity of 16 mS/cm) than in-situ. For both calibration curves the offsets were close to 0 A/m^2^ while the slopes were different (Fig. 13A). The experiment was not conducted with the large sensor.

Another experiment showed that the sensor measured the current density of the synthetic nitrite solution with a downward drift over the measurement time of 400 min while the pH and the dissolved oxygen concentration stayed nearly stable (Fig. 13B). This suggests that it takes at least 400 min to form an equilibrium between reaction kinetics, diffusion and electric field. This supports our recommendation to calibrate the sensor in-situ.

## 4 Discussion

### 4.1 Influence of environmental conditions

We found that the effects of temperature, pH, aeration, electric conductivity and dissolved oxygen concentration on the amperometric nitrite sensor are negligible. In the assessed range of temperature and pH (22.5 – 26.5 °C and 6.0 – 7.1, respectively), no significant influence was detected. Furthermore, we did not find any dependency on the aeration rate, as long as the urine nitrification reactor was aerated. Neither did a variation in electric conductivity cause any change in the sensor signal at 10 mS/cm or more. The amperometric nitrite sensor is well suited for urine nitrification, since pH, temperature and the electric conductivity are usually in ranges for which we did not find any significant influence.

### 4.2 Drift

While there was no noticeable drift in the ex-situ experiments, we observed that the amperometric nitrite sensor has a drift in-situ, i.e. the sensitivity varies while the offset stays rather stable around 0 A/m^2^. Further research is necessary in order to find out whether the sensitivity decreases steadily or whether a seasonal variability can be observed. Nevertheless, we propose that the drift can be compensated for by regular calibration, for which a one-point calibration may be sufficient. We suggest monthly in-situ calibration of the amperometric nitrite sensor. Another option is to quantify and include the drift in process control. Thürlimann et al. (2019) showed that a nitrite sensor prone to offset drift can be used for stabilizing control in urine nitrification by applying qualitative trend analysis. Note that the amperometric measurement exhibits drift of the sensitivity, which one may not be able to account for with qualitative trend analysis.

### 4.3 Accuracy of the amperometric nitrite sensor

The calibration curves of the in-situ experiments for each month resulted in a coefficient of variation of the method V_xo_, that is the standard deviation of the method s_xo_ divided by the mean concentration, of 17% or less for the nitrite concentration range of 0–50 mg_N_/L. In comparison, according to Hach Lange GmbH, spectrophotometric cuvette tests have a V_xo_ of 3% for the range of 0.6–6.0 mg_N_/L (LCK 342, Hach Lange GmbH, Germany) and for the range of 0.015–0.6 mg_N_/L (LSK 341, Hach Lange GmbH, Germany). As expected, the amperometric nitrite sensor signal is not as accurate as lab-based measurements. However, the sensor is especially valuable as an automated on-line measurement.

### 4.4 Strengths and weaknesses of sensors for nitrite measurement

To our knowledge, there exist three measurement principles that have been tested for on-line nitrite measurement in wastewater. Two of these are available commercially in the form of nitrite analyzers (colorimetric measurement principle) and spectrophotometric sensors (light absorbance measurement principle). A third principle, amperometric measurement, is tested for the first time in this work. Analyzers have the particular advantage of providing measurements that can be unbiased, precise, and drift-free. One disadvantage is that this measurement is produced ex-situ with a measurement cycle that lasts 10–20 min, thus leading to delay that may be significant for process control purposes. This also means the measurement frequency is fairly low. Even more important is that significant maintenance efforts are required for this type of equipment. This includes upkeep of the sample preparation system (e.g., filter replacement) and ensuring that reagents are both fresh and available in sufficient amounts. In contrast, spectrophotometric instruments can be used in-situ, do not require sample preparation, and can be equipped with self-cleaning devices, such as pressured air nozzles, brushes, or wipers. This reduces the cost of maintenance, while not eliminating it entirely. A key advantage of spectrophotometric measurements is that they are sensitive to many compounds. This however induces a lack of specificity, which is accounted for by specialized knowledge, such as the execution of calibration experiments and software tools for information extraction (Mašić et al., 2015; Thürlimann et al., 2019). The proposed nitrite sensor combines advantages from both analyzers and spectrophotometric measurements: it is simultaneously sensitive and specific to nitrite and is expected to require limited maintenance efforts only. In addition, it can be produced from cheap materials. The amperometric nitrite measurement is not yet a mature technology and therefore further testing, including sensor and model validation, is necessary. The observed drift of the sensor’s specificity in in-situ deployment is the only known detractor at this moment, which needs to be quantified and addressed in future research.

### 4.5 Application

We found that 90% of the data above 5 mg_N_/L have a maximum relative deviation of 20% and 21% for the large and the small sensor, respectively. The treatment of municipal wastewater requires a precise nitrite control at low concentrations, otherwise we risk nitrous oxide production (Wunderlin et al., 2012) as well as exceedance of the effluent discharge limit of 0.3 mg_N_/L for the nitrite concentration (Gujer, 2006). Since the relative prediction residuals increase strongly below 5 mg_N_/L (see section 3.2), we do not recommend the sensors in the current configuration for nitrite measurement in municipal wastewater treatment, where nitrite concentrations below 5 mg_N_/L can already be critical.

Contrary, in urine nitrification higher nitrite concentrations are tolerated. Our calculations suggest that nitrite concentrations of e.g. 12 mg_N_/L at a pH of 6.0 or 30 mg_N_/L at a pH of 6.8 must not be exceeded, in order to prevent nitrite accumulation. Based on our experience with the sensor, we assume that with the amperometric nitrite sensor, high nitrite concentrations can be avoided without difficulty. Therefore, we conclude that the sensor is a promising tool for process control of urine nitrification.

## 5 Conclusions

• With standard deviations below 4 mg_N_/L and a minimum linear nitrite range of 0–50 mg_N_/L, the amperometric nitrite sensor covers the critical range of nitrite accumulation and is well suited for on-line nitrite monitoring and process control in urine nitrification.
• We expect that the current amperometric sensor is also well suitable for controlling nitrification of other high strength nitrogen solutions such as digester supernatant. However, an increase of the sensitivity is necessary for nitrite control in the mainstream of municipal wastewater treatment.
• Drift corrections are necessary, but monthly intervals for calibration are sufficient. We propose that a one-point calibration of the sensitivity is suitable.
• The sensors must be calibrated in-situ. In urine nitrification, the increased nitrite concentrations required for the one-point calibration can be triggered by short increases of the inflow.

## Declaration of competing interest

The authors declare the following financial interests/personal relationships which may be considered as potential competing interests: Kai M. Udert is co-owner of the Eawag spin-off Vuna Ltd, which has a license for electrochemical nitrite removal and control. Peter Schrems produced the small nitrite sensor.
