# Supplementary material for: Electrochemical nitrite sensing for urine nitrification
Source: Water Res X. 2020 May 23;9:100055. doi: 10.1016/j.wroa.2020.100055 (PMC7287277; doi:10.1016/j.wroa.2020.100055)
Supplement: Multimedia component 1 [file mmc1.pdf]

Supporting Information for

Electrochemical nitrite sensing for urine nitrification

Livia Britschgi[a], Kris Villez[a], Peter Schrems[b1], Kai M. Udert[a,c,*]

[a] Eawag, Swiss Federal Institute of Aquatic Science and Technology, 8600 Dübendorf, Switzerland

[b] IPS Elektroniklabor GmbH & Co. KG, 64839 Münster, Germany

[c] ETH Zürich, Institute of Environmental Engineering, 8093 Zürich, Switzerland

* Corresponding author:

Kai M. Udert
Kai.Udert@eawag.ch
Eawag: Swiss Federal Institute of Aquatic Science and Technology
8600 Dübendorf, Switzerland

[1] Current address: ORNL, Oak Ridge National Laboratory, Oak Ridge, TN 37830, USA

S1 MATERIALS AND METHODS

S1.1 Working range

S1.1.1 Theoretical approach

In order to calculate the nitrite concentration at which the NOB activity is at its maximum, we followed the approach of Fumasoli (2016) who used Monod kinetics and the assumptions of Pambrun et al. (2006) to model the growth rate of the NOB (Equation S1). Since the urine nitrification reactor was permanently aerated at a sufficient rate, the oxygen limitation term was neglected. Furthermore, the term of HNO_2 inhibition was included because contrary to the assumption of Pambrun et al. (2006) urine nitrification is operated at a pH lower than 7, hence, HNO_2 inhibition is relevant. The kinetic parameters were assessed by Reimann (2019).

$$\frac{dX_{NOB}}{dt} = \mu_{max} * \frac{K_{I,HNO_2}}{[HNO_2]+K_{I,HNO_2}} * \frac{[NO_2^-]}{[NO_2^-]+K_{S,NO_2^-}} * \frac{K_{I,NH_3}}{[NH_3]+K_{I,NH_3}} * X_{NOB} \quad \textbf{(S1)}$$

$\frac{dX_{NOB}}{dt} =$ NOB growth rate $[g_{COD}/(L*d)]$

$\mu_{max} =$ maximum growth rate $[1/d]$

$K_{I,HNO_2} =$ constant for non-competitive inhibition by HNO_2 (= 0.138, Reimann, 2019) $[mg_N/L]$

$K_{S,NO_2^-} =$ affinity constant for NO_2^- (= 1.46, Reimann, 2019) $[mg_N/L]$

$K_{I,NH_3} =$ constant for non-competitive inhibition by NH_3 (= 72.5, Reimann, 2019) $[mg_N/L]$

$X_{NOB} =$ biomass concentration of NOB $[g_{COD}/L]$

We defined $[HNO_2]$ according to the acidic equilibrium in dependency of the nitrite activity, pH and the dissociation constant for nitrite at a temperature of 25°C (Equation S2) (Tchobanoglous et al., 2014) and used the term in Equation S1.

$$\{HNO_2\} = f_{A,HNO_2} * [HNO_2] = [HNO_2] = \frac{\{NO_2^-\}*\{H^+\}}{K_{NO_2^-}} = \frac{f_{A,NO_2^-}*[NO_2^-]*10^{-pH}}{10^{-pK_{NO_2^-}}} \quad \text{(S2)}$$

$\{...\} =$ **activity** $[-]$

$[...] =$ **concentration** $[mg_N/L]$

$f_{A,HNO_2} =$ activity coefficient for HNO_2 (= 1) $[-]$

$f_{A,NO_2^-} =$ activity coefficient for NO_2^- (= 0.75) $[-]$

$K_{NO_2^-} =$ acid dissociation constant for NO_2^-
(= $10^{-3.29}$ at 25°C, Schwartz & White, 1981) $[-]$

The activity coefficient for nitrite was calculated with the Davies approach (Stumm & Morgan, 1996) in dependency of the ionic strength (Equation S3). The ionic strength was determined according to Equation S4 considering the main inorganic ions, ammonium, potassium, sodium, nitrate, chloride, sulfate and phosphate, for pH 6.0 to 6.8 (Table S1).

$$log_{10}(f_A) = -A * Z_i^2 * \left(\frac{\sqrt{I}}{1+\sqrt{I}} - 0.2 * I\right) \quad \text{(S3)}$$

$$I = \frac{1}{2} * \sum_i C_i * Z_i^2 \quad \text{(S4)}$$

$A \approx$ 0.5 for water at 25°C $[-]$

$Z_i =$ charge of ion $[-]$

$I =$ ionic strength of solution $[mol/L]$

Table S1. Ionic strength (based on Pitzer approach) and activity coefficient (based on Davies approach) in dependency of the pH.

pH [-]	Ionic strength I [mol/L]	Activity coefficient f_{A,NO_2^-} [-]
6.0	0.159	0.747
6.1	0.160	0.747
6.2	0.160	0.747
6.3	0.160	0.747
6.4	0.160	0.747
6.5	0.161	0.746
6.6	0.161	0.746
6.7	0.161	0.746
6.8	0.162	0.746

In order to determine the maximum growth rate, we derived Equation S1 after $[NO_2^-]$ and equalized it to 0 (Equation S5).

$$\frac{d\left(\frac{dX_{NOB}}{dt}\right)}{d[NO_2^-]} = 0 \quad \textbf{(S5)}$$

We solved the resulting equation after $[NO_2^-]$. The resulting nitrite concentration $[NO_2^-]_{opt}$ in dependency of the pH at which the NOB growth rate is at its maximum is shown in Equation S6.

$$[NO_2^-]_{opt} = \sqrt{\frac{K_{I,HNO_2} * K_{S,NO_2^-} * K_{NO_2^-} * 10^{pH}}{f_{A,NO_2^-}}} \quad \textbf{(S6)}$$

S1.1.2 Practical approach

Additionally, we estimated the nitrite concentration for maximum NOB activity based on a practical approach. We used the data of Thürlimann et al. (2019) and repurposed it to obtain a ballpark estimate of the nitrite concentration at which NOB exhibit maximum activity.

Figure S1 shows the raw total nitrite-nitrogen (TNN) concentration data. This includes (a) a sensor signal obtained by soft-sensing nitrite on the basis of UV-Vis light absorbance spectra recorded with an S::CAN spectro::lyser and S::CAN con::stat, (b) two laboratory measurements (Dr. Lange), and (c) one strip test. The selected data was one of the few events where (i) the maximum nitrite concentration was significantly higher than the targeted nitrite concentration, (ii) an inflection point was clearly visible, and (iii) a number of non-zero and zero-valued nitrite concentration values have been recorded. In addition, the treated volume of urine was close to linear during this time, which suggests that the activity of the ammonia oxidizing nitrifying bacteria (AOB) was constant during this time.

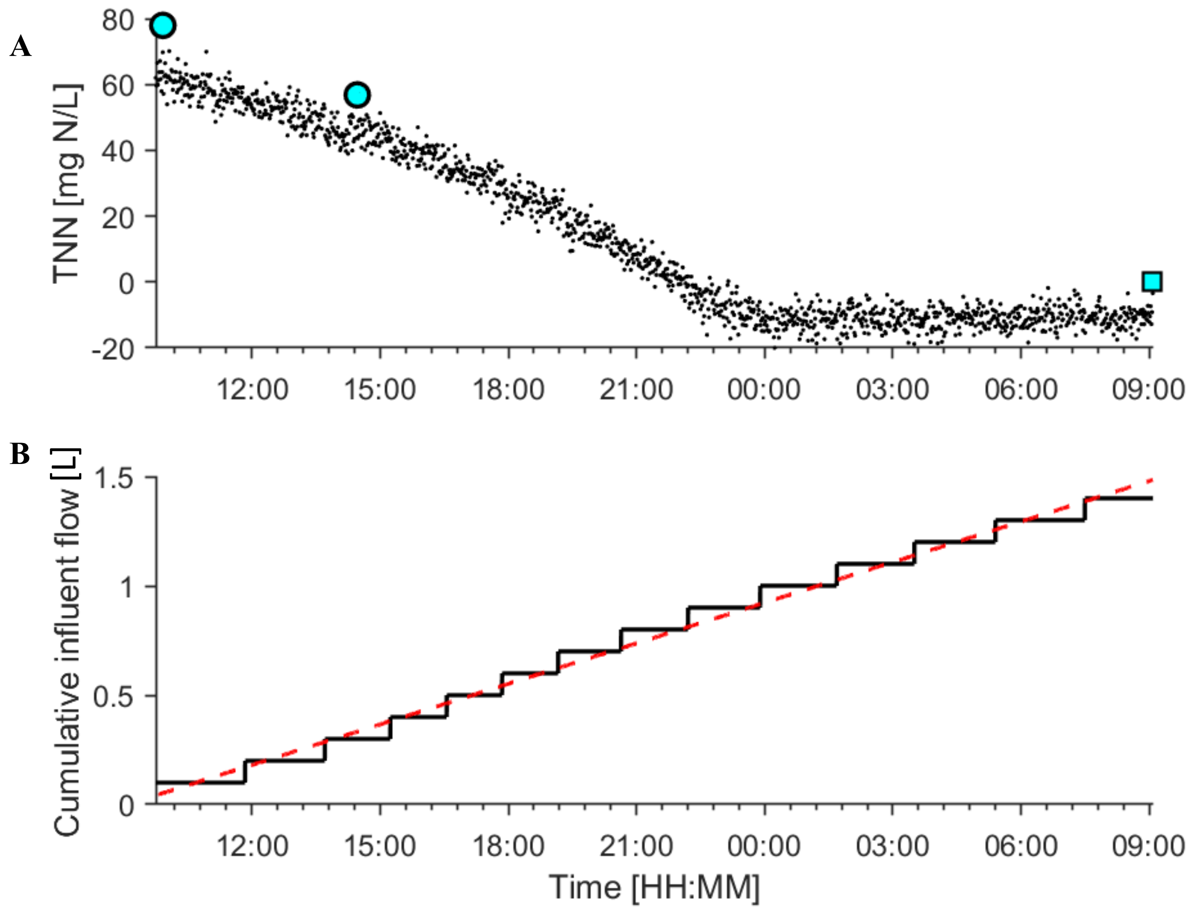

Figure S1. (A) Black dots - sensor signal; blue circles - Dr Lange; blue square - strip test. (B) Black line - treated volume of urine; red line – linear approximation.

We fitted a shape-constrained spline function with a shape consisting of two segments (episodes). The first episode had a decreasing and concave shape (primitive=D) while the second episode had a decreasing and convex shape (primitive=A). The point between the two primitives is known as an inflection point and its exact location was estimated during fitting. The chosen spline function was piece-wise cubic polynomials (polynomial degree =3) with each polynomial piece covering 64 consecutive data points (approx. 1h of data). This choice leads to a smooth yet well-fitting curve as shown in Figure S2.

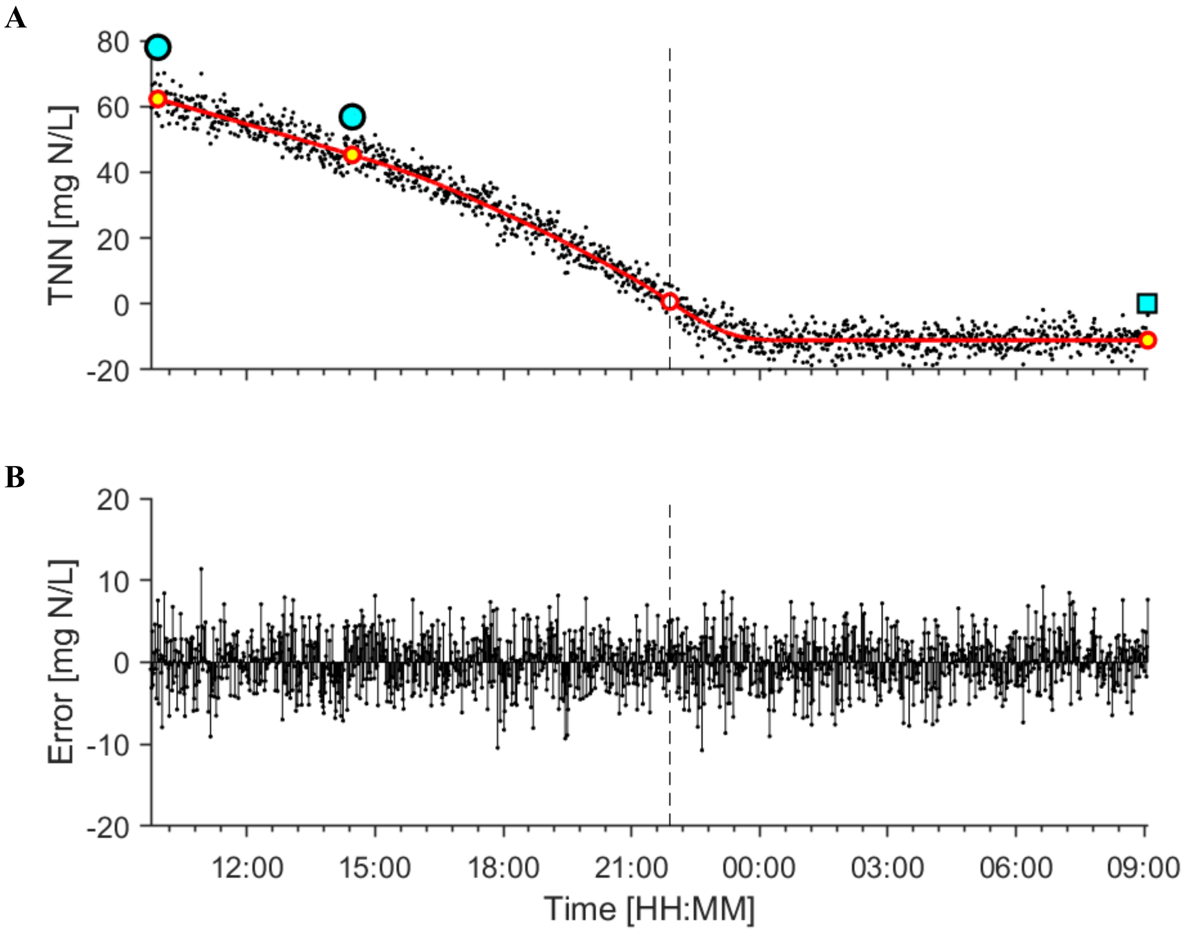

Figure S2. (A) Black dots - sensor signal; blue circles - Dr Lange; blue square - strip test; red line: shape constrained spline function. (B) Residuals between sensor signal and spline function.

The estimated inflection point was located shortly after 21:00. At this time, the fitted signal value was 0.70 mg_N/L. Note however that the produced sensor signal and the fitted curve both deviated substantially from the recorded laboratory measurements and the strip test value. This is addressed next.

The sensor signal was modified by means of local calibration. To this end, the final spline function value in Figure S2 (-11.0467 mg_N/L) was assumed to correspond to a zero-valued concentration for nitrite. This enabled an offset correction by simply subtracting this final value from the complete curve. The two laboratory measurements were then used to multiply this new signal by a slope coefficient such that the fitted curve matched these laboratory measurements best in the least-squares sense. The result of these modifications is shown in Figure S3. After these modifications, the nitrite value of the fitted curve at the inflection point equals 12.25 mg_N/L.

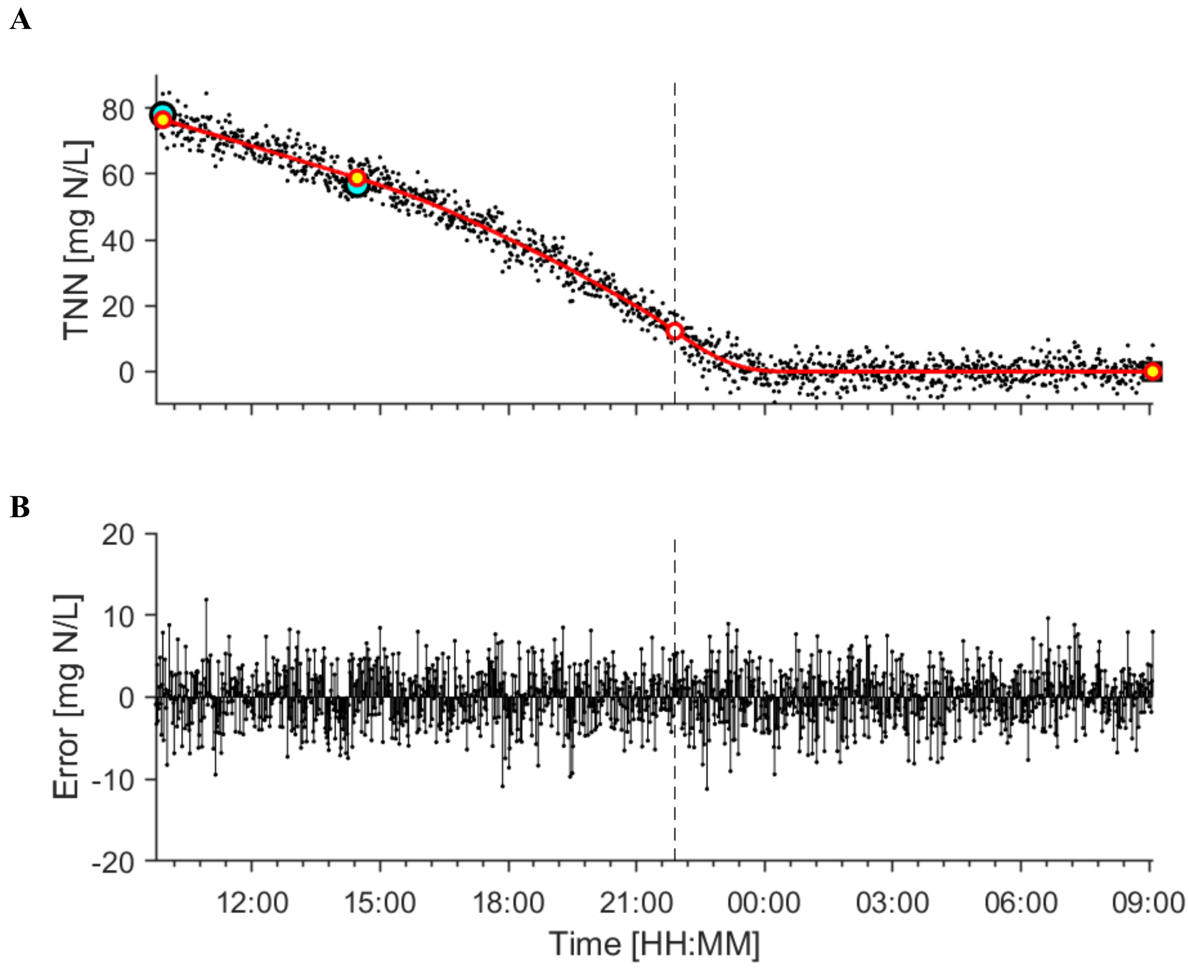

Figure S3. (A) Black dots - sensor signal; blue circles - Dr Lange; blue square - strip test; red line: locally calibrated shape constrained spline function. (B) Residuals between sensor signal and spline function.

We conclude that the maximum activity of the NOB is obtained at approximately 12 mg_N/L at a pH between 5.9 and 6.1. This estimate is considered a ballpark value as:

1. It was based on a single event
2. It assumed – within reason – that the AOB activity was constant during the event.
3. It assumed – within reason – that shape constrained spline function was a good approximation of the true nitrite concentration profile.

S1.2 Model structure identification

The identified model was obtained by selecting a parsimonious model among a large number of polynomial regression models. Table S2 shows the definition of the variables that were considered as model inputs. For computational purposes, the measured values of these variables were mean-centered and scaled to unit variance as described in Equations S7 to S9.

Table S2: Parameter definition for the model structure identification.

Parameter #	Parameter
1	1 (offset)
2	$[NO_2\text{-}N]$
3	$[NO_2\text{-}N]^2$
4	$[NO_2\text{-}N]^3$
5	T
6	T^2
7	T^3
8	pH
9	pH^2
10	pH^3

$$\mu_p = \frac{\sum_{i=1}^{n} p_i}{n} \quad \textbf{(S7)}$$

$$\sigma_p = \sqrt{\frac{\sum_{i=1}^{n}(p_i-\mu_p)^2}{n}} \quad \textbf{(S8)}$$

$$\overline{p_i} = \frac{p_i-\mu_p}{\sigma_p} \quad \textbf{(S9)}$$

p_i = parameter to be scaled

n = number of values of the parameter

μ_p = mean of all values of the parameter

σ_p = standard deviation of the parameter

$\overline{p_i}$ = scaled parameter

Equation S10 shows the formula used in the polynomial regression in a general form where j is the current density. In Equation S11 the values used in the present assessment were inserted. ε was assumed to be zero and Equation S11 was solved to β which has the form shown in Equation S12. With β, the electric current was described in dependency of nitrite, temperature and pH (Equation S13).

$$\boldsymbol{j} = \boldsymbol{x} * \boldsymbol{\beta} + \boldsymbol{\varepsilon} \tag{S10}$$

$$\begin{pmatrix} j_1 \\ \vdots \\ j_n \end{pmatrix} = \begin{pmatrix} 1 & [NO_2^-]_1 & [NO_2^-]_1^2 & [NO_2^-]_1^3 & T_1 & T_1^2 & T_1^3 & pH_1 & pH_1^2 & pH_1^3 \\ \vdots & \vdots & \vdots & \vdots & \vdots & \vdots & \vdots & \vdots & \vdots & \vdots \\ 1 & [NO_2^-]_n & [NO_2^-]_n^2 & [NO_2^-]_n^3 & T_n & T_n^2 & T_n^3 & pH_n & pH_n^2 & pH_n^3 \end{pmatrix} * \boldsymbol{\beta} + \boldsymbol{\varepsilon} \tag{S11}$$

$$\boldsymbol{\beta} = \begin{pmatrix} \beta_0 \\ \beta_{[NO_2^-]} \\ \beta_{[NO_2^-]^2} \\ \beta_{[NO_2^-]^3} \\ \beta_T \\ \beta_{T^2} \\ \beta_{T^3} \\ \beta_{pH} \\ \beta_{pH^2} \\ \beta_{pH^3} \end{pmatrix} \tag{S12}$$

$\beta_0 =$ $[A/m^2]$

$\beta_{[NO_2^-]} =$ $[(A/m^2)/(mg_N/L)]$

$\beta_{[NO_2^-]^2} =$ $[(A/m^2)/(mg_N/L)^2]$

$\beta_{[NO_2^-]^3} =$ $[(A/m^2)/(mg_N/L)^3]$

$\beta_T =$ $[(A/m^2)/(°C)]$

$\beta_{T^2} =$ $[(A/m^2)/(°C)^2]$

$\beta_{T^3} =$ $[(A/m^2)/(°C)^3]$

$\beta_{pH} =$ $[A/m^2]$

$\beta_{pH^2} =$ $[A/m^2]$

$\beta_{pH^3} =$ $[A/m^2]$

$$\boldsymbol{j} = \beta_0 + \beta_{[NO_2^-]} * [NO_2^-] + \beta_{[NO_2^-]^2} * [NO_2^-]^2 + \beta_{[NO_2^-]^3} * [NO_2^-]^3 + \beta_T * T + \beta_{T^2} * T^2 + \beta_{T^3} * T^3 + \beta_{pH} * pH + \beta_{pH^2} * pH^2 + \beta_{pH^3} * pH^3 \tag{S13}$$

In order to test which of the parameters shown in Table S2 improve the model all possible combinations of these ten parameters were analysed. This resulted in 1023 different models including one to ten parameters (variable x in Equation S10 had therefore between one and ten columns).

In a next step, the data were split into two groups according to the venetian blinds principle to conduct a cross-validation, i.e. every 1st, 3rd, etc. sample belongs to Group A and every 2nd, 4th, etc. sample belongs to Group B. This principle was applied, instead of splitting the data into half according to the time of its sampling, in order to have an even mix of different nitrite concentrations in both groups. Using this method, a drift of the sensor would not be recognized. However, a frequent ex-situ validation of the sensor was conducted, where such a drift would be noticed.

The cross-validation was conducted with two groups as follows:

1. The polynomial regression was conducted with Group A.
2. The assessed models were tested with Group B.
3. The root mean square error (RMSE) of Group B was calculated for each model (see Equation S14).
4. The polynomial regression was conducted with Group B.
5. The assessed models were tested with Group A.
6. The root mean square error (RMSE) of Group A was calculated for each model (see Equation S14).
7. The mean RMSE of Group A and Group B was calculated to determine the suitability of each model.
8. The models were compared according to their mean RMSE.

The RMSE was calculated according to Chai and Draxler (2014), as shown in Equation S14.

$$\boldsymbol{RMSE} = \sqrt{\frac{1}{n}\sum_{i=1}^{n}(p_i - \hat{p_i})^2} \qquad \textbf{(S14)}$$

$p_i =$ measured value

$\hat{p_i} =$ estimated value

$n =$ number of measured values

Some of the models resulted in imaginary values for the nitrite concentration. In the present analysis the imaginary values were neglected.

S1.3 Temperature and pH

While assessing the influence of temperature and pH on the amperometric nitrite sensor, we collected 79 random samples between 0 and 50 mg_N/L at 22.5 to 26.5°C and at pH 6.0 to 7.1 with the large sensor, shown in Figure S4A. With the small sensor, we collected 31 random samples between 0 and 50 mg_N/L at 23.1 to 26.4°C and at pH 6.0 to 7.0, shown in Figure S4B.

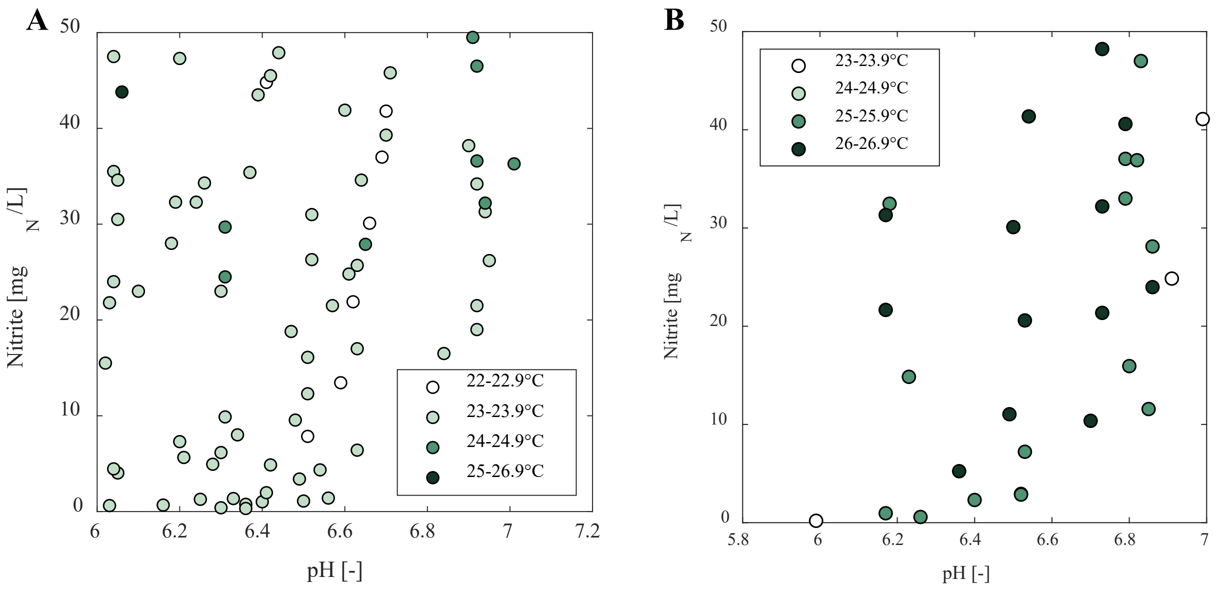

Figure S4. Nitrite concentration of all samples taken during the temperature and pH dependency assessment with the corresponding temperatures and pH values. (A) The measurements of the large sensor and (B) of the small sensor.

S1.4 Response time

The assessment of the response time of the amperometric nitrite sensor was done on the basis of ISO 15839 (ISO, 2003). In this initial experiment, we considered the working range to be 0 to 24 mgN/L. Therefore, the response time assessed in this study is only valid for that working range. The experiment was conducted as follows:

- Two stock solutions at 20% and 80% of the working range were produced: c_{20} = 4.8 mg_N/L and c_{80} = 19.2 mg_N/L at an electric conductivity of 16 mS/cm as in nitrified urine (Etter et al., 2013) with nanopure water, $NaNO_2$ and NaCl (assay = 99% and ≥ 99.5%, respectively, Merck KGaA, Germany).
- 250 mL of the stock solutions were put into a glass beaker and continuously stirred with a magnetic stirrer at 600 rpm (color squid white, IKA®, Germany), each.
- The sensor was mounted on a stand to ensure a fixed position during the time the sensor was submerged in one of the solutions.
- The sensor was then put into the solutions alternately, 1.5 minutes at a time. Contrary to ISO 15839 (ISO, 2003), the change between the two solutions was not immediate but was done manually and therefore took several seconds.

The data was interpreted as follows:

- We transformed the sensor signal to percentage, where the mean value of the signal at c20 equals 0% and the mean value of the signal at c80 equals 100%.
- Rise time: T10 is the time at which 10% of the signal increase caused by the change of the stock solution is reached, T90 accordingly (Figure S5A).
- Fall time: T10 is the time at which 10% of the signal decrease caused by the change of the stock solution is reached, T90 accordingly (Figure S5B). The fall time includes the time it took to switch from the higher concentrated to the lower concentrated stock solution and hence is an overestimation of the actual fall time.
- Rise time resp. fall time = T90 – T10.
- The mean rise and fall time of 10 runs was calculated.

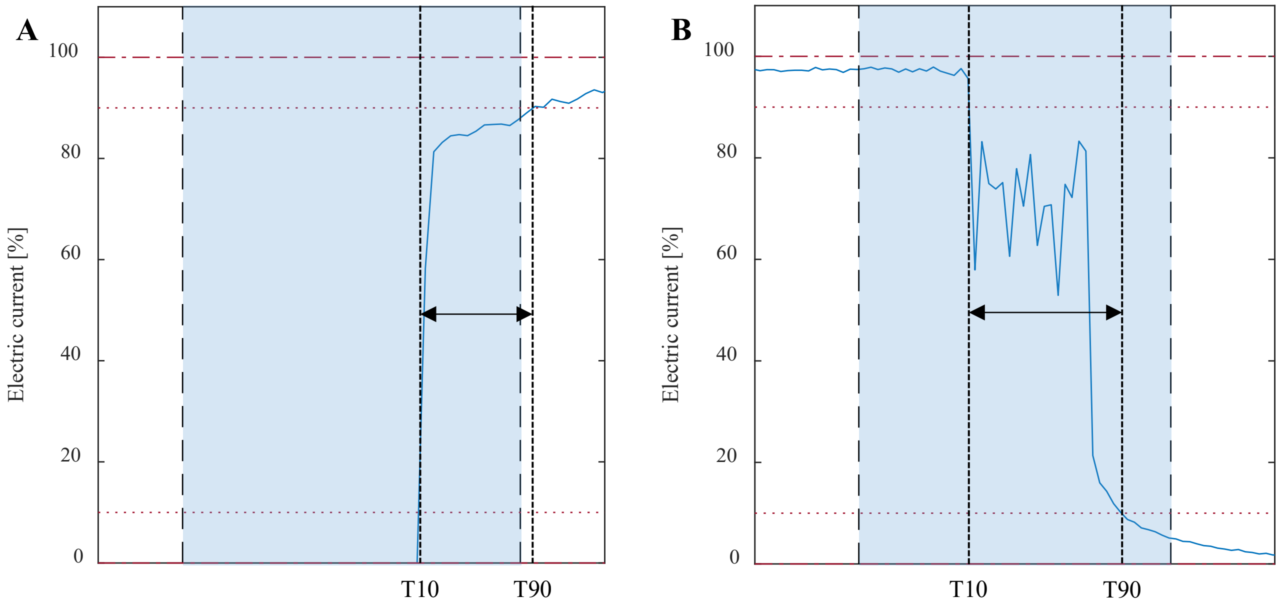

Figure S5. Response of the signal of the amperometric nitrite sensor on a concentration increase (A) and a concentration decrease (B). The blue square indicates the time during which the sensor was changed from one stock solution to the other.

S2 RESULTS

S2.1 Data calibration

For each month, we fitted a linear curve for the data between 0 and 50 mg_N/L with a least squares fit (Table S3). The measurements from February 2019 resulted in a large 95%-confidence interval. In this month, we collected three samples only, of which one was collected 2 weeks after the other two samples.

Table S3. Slope, offset, variation of the method V_{xo} and the maximum 95%-confidence interval of monthly separated data from the large and small sensor. For each month, a linear curve was fitted.

	**Slope [$(A/m^2)/(mg_N/L)$]**	**Offset [A/m^2]**	**V_{xo} [%] = s_{xo}/mean**	**Max. 95%-confidence interval [mg_N/L]**
Large sensor				
April 2018	0.3084	0.2051	14.6	±6.3
May 2018	0.2827	0.2653	13.8	±7.2
June 2018	0.4145	1.2237	12.8	±6.8
July 2018	0.3897	0.7736	3.7	±5.7
Small sensor				
October 2018	0.1453	0.1451	13.3	±6.4
November 2018	0.1812	0.0007	10.2	±5.9
January 2019	0.0996	0.2075	17.2	±9.3
February 2019	0.0456	0.2850	11.6	±45.9

S2.2 Temperature and pH

Figure S6 shows the results of the temperature and pH dependency assessment conducted with the small sensor, which date from October 2018 to January 2019. The linear model (Model B) shown in Figure S6A results in an RMSE of 0.6 A/m^2. Model A which best describes the data (current density as a function of an offset, [NO_2^-], [NO_2^-]^2, [NO_2^-]^3, T, T^2, T^3, pH, pH^2, pH^3) results in an RMSE of 0.3 A/m^2. This confirms the results from the assessment with the large sensor. Model B results in a standard deviation of the method s_{xo} of 4.4 mg_N/L.

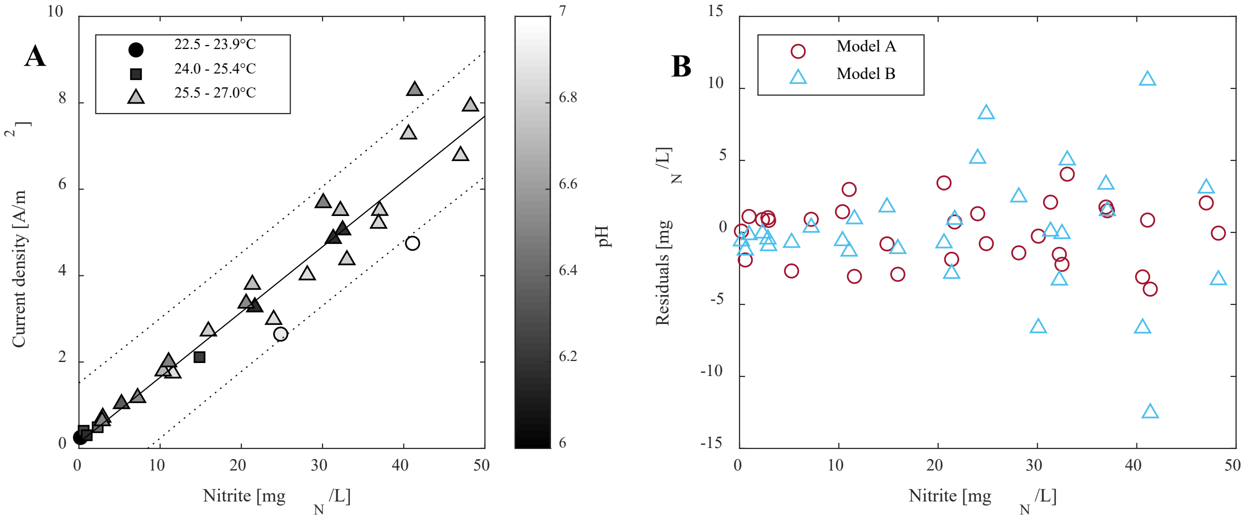

Figure S6. (A) Linear curve and the corresponding 95%-confidence interval describing the current density in dependency of nitrite, neglicting temperature and pH (Model B).
(B) Comparison of the residuals of the model best describing the correlation (Model A: including nitrite, temperature and pH) and the linear model only depending on nitrite (Model B). The results were obtained with the small sensor.

S2.3 Aeration

Figure S7 shows the results of the aeration dependency assessment conducted in November 2018 and January 2019 with the small sensor. The results from the analysis conducted with the large sensor could be confirmed. The linear curve results in a standard deviation of the procedure s_{xo} of 4.2 mg_N/L.

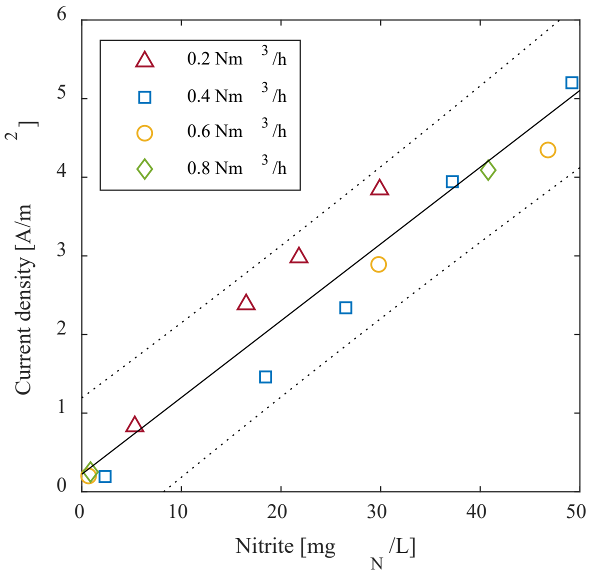

Figure S7. Samples of the aeration dependency assessment with the fitted linear curve with the corresponding 95%-confidence interval. The results were obtained with the small sensor.

S2.4 Typical wear-and-tear

In Figure S8A, B and C the results of the fouling experiment, conducted with the large sensor with artificial nitrite solutions and ex-situ, are depicted. The results from the ex-situ assessment with the small sensor and nitrified urine are shown in Figure S8D.

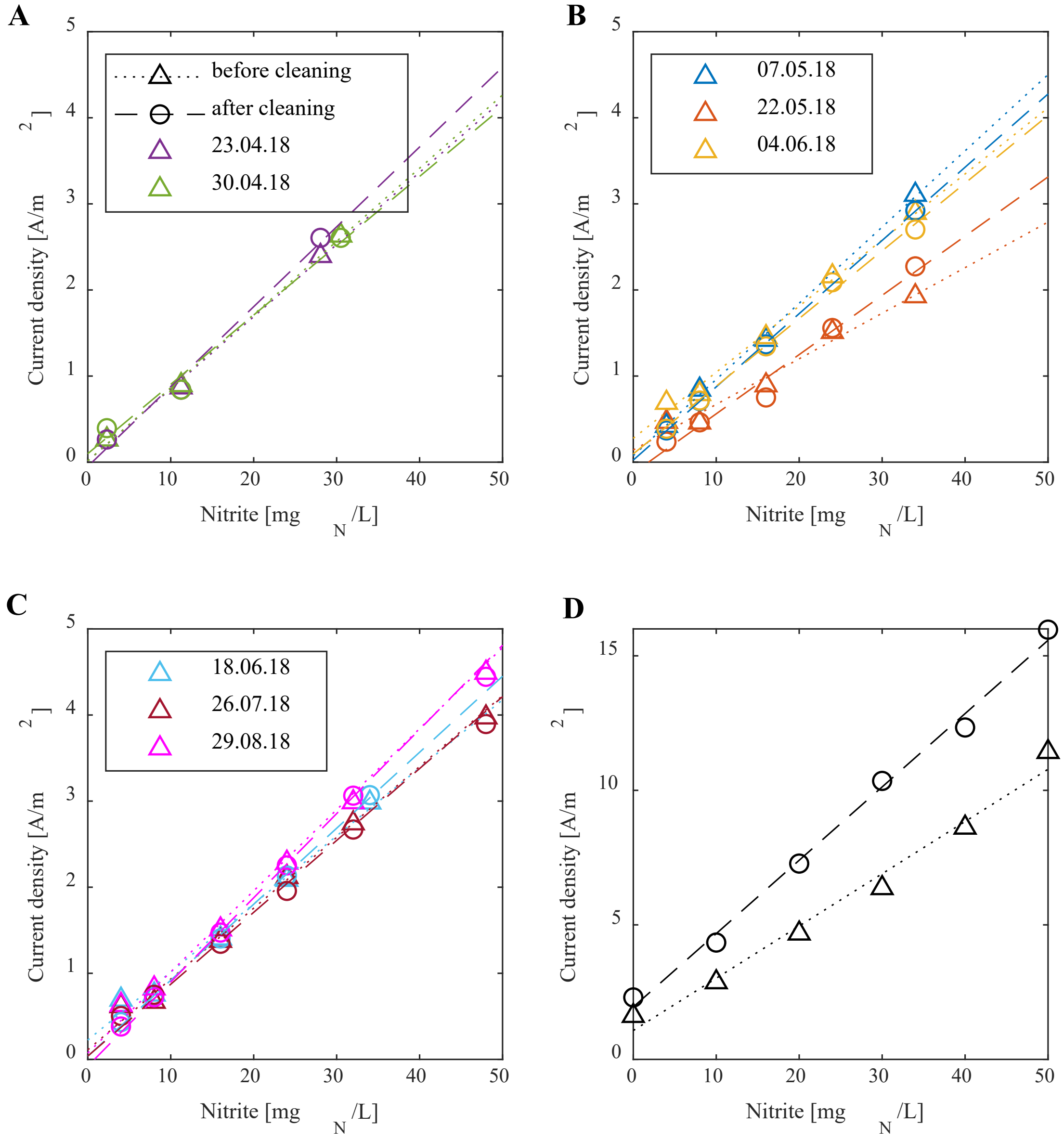

Figure S8. (A), (B) and (C) Results from the ex-situ fouling analysis with artificial nitrite solutions. The measurements were conducted with the large sensor. (D) Results from the ex-situ fouling analysis with nitrified urine. The measurements were conducted with the small sensor after it was within the nitrification reactor for 10 days.

S3 REFERENCES

Chai, T., & Draxler, R. R. (2014). Root mean square error (RMSE) or mean absolute error (MAE)? – Arguments against avoiding RMSE in the literature. *Geosci. Model Dev., 7*(3), 1247-1250. doi:10.5194/gmd-7-1247-2014

Etter, B., Hug, A., & Udert, K. M. (2013). *Total Nutrient Recovery from Urine – Operation of a Pilot-Scale Nitrification Reactor*. Retrieved from Dübendorf, Switzerland: https://www.researchgate.net/publication/265966790_Total_Nutrient_Recovery_from_Urine_-_Operation_of_a_Pilot-Scale_Nitrification_Reactor

Fumasoli, A. (2016). *Nitrification of Urine as Preatreatment for Nutrient Recovery.* (Doctoral Thesis), ETH Zurich, Dübendorf.

ISO. (2003). *Water quality - On-line sensors/analysing equipment for water - Specifications and performance tests*. Retrieved from Geneva, Switzerland:

Pambrun, V., Paul, E., & Spérandio, M. (2006). Modeling the partial nitrification in sequencing batch reactor for biomass adapted to high ammonia concentrations. *Biotechnology and Bioengineering, 95*(1), 120-131. doi:10.1002/bit.21008

Reimann, M. (2019). *Optimizing Urine Nitrification For Space Mission - Nitrite Oxidizing Bacteria Kinetics Calibration.* (Master Project), ETH Zürich, Zürich.

Schwartz, S. E., & White, W. H. (1981). Solubility equilibria of the nitrogen oxides and oxyacids in dilute aqueous solution. *Adv. Environ. Sci. Eng.; (United States)*, Medium: X; Size: Pages: vp.

Stumm, W., & Morgan, J. J. (1996). *Aquatic chemistry : chemical equilibria and rates in natural waters* (3rd ed. ed.): New York : Wiley.

Tchobanoglous, G., Burton, F. L., & Stensel, H. D. (2014). *Wastewater Engineering: Treatment and Resource Recovery*: McGraw-Hill.

Thürlimann, C. M., Udert, K. M., Morgenroth, E., & Villez, K. (2019). Stabilizing control of a urine nitrification process in the presence of sensor drift. *Water Research, 165*, 114958. doi:https://doi.org/10.1016/j.watres.2019.114958
